# Past and Ongoing Tsetse and Animal Trypanosomiasis Control Operations in Five African Countries: A Systematic Review

**DOI:** 10.1371/journal.pntd.0005247

**Published:** 2016-12-27

**Authors:** Anne Meyer, Hannah R. Holt, Richard Selby, Javier Guitian

**Affiliations:** 1 Department of Production and Population Health, Royal Veterinary College, Hatfield, United Kingdom; 2 Department of Vector Biology, Liverpool School of Tropical Medicine, Liverpool, United Kingdom; Hunter College, CUNY, UNITED STATES

## Abstract

**Background:**

Control operations targeting Animal African Trypanosomiasis and its primary vector, the tsetse, were covering approximately 128,000 km^2^ of Africa in 2001, which is a mere 1.3% of the tsetse infested area. Although extensive trypanosomiasis and tsetse (T&T) control operations have been running since the beginning of the 20^th^ century, Animal African Trypanosomiasis is still a major constraint of livestock production in sub-Saharan Africa.

**Methodology/Principal Findings:**

We performed a systematic review of the existing literature describing T&T control programmes conducted in a selection of five African countries, namely Burkina Faso, Cameroon, Ethiopia, Uganda and Zambia, between 1980 and 2015. Sixty-eight documents were eventually selected from those identified by the database search. This was supplemented with information gathered through semi-structured interviews conducted with twelve key informants recruited in the study countries and selected based on their experience and knowledge of T&T control. The combined information from these two sources was used to describe the inputs, processes and outcomes from 23 major T&T control programmes implemented in the study countries. Although there were some data gaps, involvement of the target communities and sustainability of the control activities were identified as the two main issues faced by these programmes. Further, there was a lack of evaluation of these control programmes, as well as a lack of a standardised methodology to conduct such evaluations.

**Conclusions/Significance:**

Past experiences demonstrated that coordinated and sustained control activities require careful planning, and evidence of successes, failures and setbacks from past control programmes represent a mine of information. As there is a lack of evaluation of these programmes, these data have not been fully exploited for the design, analyses and justification of future control programmes.

## Author Summary

Despite decades of control efforts targeting Animal African Trypanosomiasis, its distribution has hardly receded and the situation in sub-Saharan Africa has recently been described as “serious and deteriorating”. This disease of livestock affects farmers’ livelihood and welfare, food security and poses a threat to public health. Our work was primarily based on a review of available information on present and past control programmes targeting Animal African Trypanosomiasis and its vector, the tsetse, supplemented with interviews with key informants. We described and contextualised 23 well documented programmes implemented in five countries (Burkina Faso, Cameroon, Ethiopia, Uganda and Zambia). We summarised and discussed the issues commonly faced by these programmes and identified settings and circumstances which are more favourable for successful control activities. This work can contribute to the design and implementation of future programmes by highlighting the knowledge gained from past activities and experiences of those involved, and making recommendations as to how this can be utilised further.

## Introduction

Animal African Trypanosomiasis (AAT) and its primary vector, the tsetse, are among the biggest constraints to sustainable livestock production in Africa [1]. Although extensive trypanosomiasis and tsetse (T&T) control operations have been running since the beginning of the 20^th^ century, tsetse infestation in sub-Saharan Africa has hardly receded. Data provided by Allsopp [2] suggest that vector control operations were covering approximately 128,000 km^2^ of Africa in 2001, a mere 1.3% of the tsetse infested area. In areas without effective vector control, trypanocides are widely used to control AAT in cattle. However, no new veterinary drugs for the treatment of AAT have been released since 1985 [3] and there is increasing resistance to the existing trypanocides. Since 2000, there has been renewed interest and funding committed to AAT control, as well as research into drug discovery and novel control methods [4]. In addition, the Pan-African Tsetse and Trypanosomiasis Eradication Campaign (PATTEC), whose coordinating office is supported by the African Union, has set tsetse and AAT elimination as its goal.

**Fig 1:**
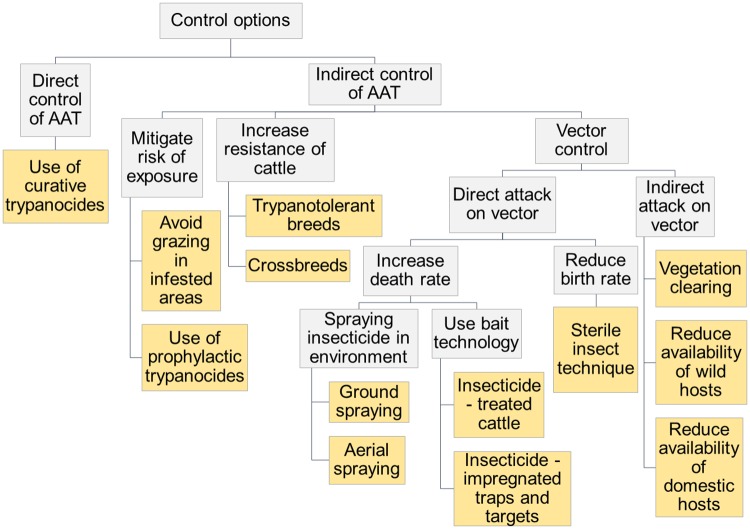
Summary diagram of the techniques available to control tsetse and AAT.

Elimination of a disease has been defined as “the reduction to zero in the incidence of a specified disease in a defined geographical area” [5]. Whether this goal is achievable in the context of AAT has been highly debated and many disease experts believe that sustained reduction in disease incidence to a locally acceptable level (“control”) is a more realistic target [6, 7]. However, geographic variation in T&T species distribution and eco-epidemiology of the disease, as well as disparities in resource distribution, infrastructure and political stability within and between sub-Saharan Africa countries raises questions as to the plausibility of long term sustainable AAT control at sub-continent level [2]. Before the 1950s, T&T control mostly involved methods with negative environmental impacts such as bush clearing, ground spraying with dichlorodiphenyltrichloroethane (DDT) and wildlife culling. From the 1980s, more ecologically and politically acceptable methods were developed such as selective bush clearing, sequential aerial spraying (SAS), insecticide-treated traps and targets (ITT), insecticide-treated cattle (ITC) used as live baits and eventually the sterile insect technique (SIT) (see [2, 8] for detailed reviews of vector control techniques). More recently, several studies showed that restricted applications of insecticides on cattle (spray on lower body parts, footbaths) were an effective cheaper control option [9–11]. An overview of available control methods is given in Fig 1.

An upsurge of AAT cases occurred in numerous places during the post-independence period, as a result of a decline in the funding available for T&T control. AAT incidence was very high in the 1980s-1990s, where cattle herds almost disappeared in some areas, such as the Ghibe Valley of Ethiopia and the Yale Province of Burkina Faso [1]. The late 1990’s saw renewed interest in the control of the disease in sub-Saharan Africa and several programmes were established to address the issue of AAT. The Programme Against African Trypanosomiasis established in 1997 and led by the Food and Agriculture Organisation includes the development of an Atlas of tsetse and AAT which aims “to build and regularly update a geospatial database of tsetse species occurrence and AAT at the continental level” [12]. The first step of the project was to collect and harmonize available data (since 1980) from peer-reviewed articles and grey literature. This program is still ongoing.

The Farming In Tsetse Controlled Areas (FITCA) programme was implemented from 1997 to 2004 in East Africa. Funded by the European Development Fund, it operated in Kenya, Uganda, Ethiopia and Tanzania. Initially it made use of impregnated targets and traps set up by government officials, followed-up with insecticide treatment of cattle. The project also encouraged the use of zero grazing dairy units protected by insecticide-treated nets [13]. As the programme emphasised the involvement of communities in T&T control, it assisted groups of farmers to implement and manage regular insecticide-treatment of cattle themselves. However, the operations were not sustained in most areas. Some results of impact assessment have been presented for Uganda, but not for Ethiopia, where the focus was mainly on capacity building in four provinces [14].

From 1986 to 1995, four Southern African countries received assistance and funding from the Regional Tsetse and Trypanosomiasis Control Programme (RTTCP) to eliminate tsetse in the region. This program, mostly funded by the European Commission, targeted the “common tsetse belt” which covered about 322,000 km^2^ in Malawi, Mozambique, Zambia and Zimbabwe [15]. Although the overall success of the project is controversial, over 20,000 km^2^ of land were cleared in Zimbabwe during that period [16, 17].

The PATTEC was initiated in 2000 during the 36^th^ meeting of the African Union. The primary objective of the campaign is to clear the African continent from tsetse flies, by using an area-wide approach. Phase I of the PATTEC campaign, named “Creation of Sustainable Tsetse and Trypanosomiasis free areas in East and West Africa”, targeted three countries in West Africa (Burkina Faso, Ghana and Mali) and three in East Africa (Ethiopia, Kenya and Uganda) [18]. The Southern Rift Valley and the West African Moist Savannah Zone were chosen by PATTEC because of their high potential for agricultural development (i.e. short-term benefits) [19].

Sub-Saharan Africa has a long and complex history of T&T control. These past and current control activities are a mine of information which should be fully exploited when designing future programmes. The outcomes obtained and the experience gained from these activities should be made available to those involved in T&T research, policy design and implementation. This review aims to identify, summarise and appraise published and unpublished information on control programs implemented in different settings. Five countries, namely Burkina Faso, Cameroon, Ethiopia, Uganda and Zambia, were selected, capturing five of the six ecosystems of inter-tropical Africa (rainforest, moist deciduous forest, mountainous, dry forest, and scrublands [20]), the sixth ecosystem being tropical desert, from which tsetse are absent. These countries represent the broad spectrum of contexts encountered by T&T interventions, in terms of trypanosomiasis epidemiology [21] and disease impact and management, as well as an overview of the diversity of the interventions themselves. This work is part of a larger project on T&T control where these countries were chosen for the implementation of “knowledge, attitudes and perceptions” surveys of farmers in relation to AAT (see also [22]). We utilised qualitative and quantitative data sources to describe and compare specific control programmes initiated in the countries of interest, extracting information on their inputs, processes and outcomes. These data were used in order to assess the success of the selected programmes in an objective manner, summarise lessons learnt during their implementation and identify whether standardised methodologies exist for their evaluation. The majority of data came from a systematic review of existing literature and this was supplemented with semi-structured interviews with experts and key actors in the field of T&T control.

## Methods

### Ethics statement

This study has been approved by the Clinical Research and Ethical Review Board of the Royal Veterinary College (project number 2504).

### Literature review

We collected existing peer-reviewed publications for the selected countries and extracted relevant information regarding AAT control and programme-specific data including outcome measures, where available. Three databases were searched in May 2015 (and repeated in May 2016) for relevant documents: ScienceDirect, Web Of Science and PubMed. The search was conducted separately for each study country, with the following key words: control AND (trypanosom* OR tsetse) AND “*country name*”. All documents published in English or French since 1980 were considered.

Titles and abstracts were screened by AM to assess the relevance of the selected documents using the following eligibility criteria: i) document related to T&T control for AAT, ii) control implemented in at least one of the five study countries, iii) control operations started after 1980. We excluded research-oriented trials which were primarily aimed at evaluating control methods rather than controlling T&T in the recipient community. We focussed this review on the control of AAT. However, a few T&T control operations targeting both AAT and HAT, in areas where livestock are a reservoir of HAT, were also included. Articles which did not pass the relevance assessment could still be used to provide a narrative of AAT control in the region. Articles which passed the primary screening as well as articles where the relevance could not be determined by reading the abstract alone were read in full. Articles were finally included if they met the above eligibility criteria. These data were then supplemented with published and unpublished literature and reports from relevant institutions sourced from Google, organisation websites, the references of identified articles and from the key informants.

### Semi-structured interviews

Upon completion of the literature review, and in order to corroborate the results, fill identified data gaps and identify control programs that had not been documented in the literature, semi-structured interviews were conducted with key informants involved in tsetse control in each of the countries studied. Potential respondents were identified by approaching contacts of the article authors, as well as individuals suggested by these contacts and individuals personally met during the course of previous T&T related research activities. A number of key informants were then selected, based either on a track record in research closely related to the T&T control programmes identified or having significant involvement in the design and delivery of these programmes, at national or international level. The telephone interviews were recorded and the relevant information was summarized by the interviewer. The interviews followed a semi-structured format, including the following sections: i) major past control operations implemented in the country since 1980 which have not been identified during the literature review, ii) involvement of the different partners (government, other institutions, non-governmental organisations, community) within T&T projects, iii) sustainability of these projects and evolution of the situation after the end of the projects, iv) progress and results of the current PATTEC campaign in the country (if applicable).

### Data extraction

**Fig 2:**
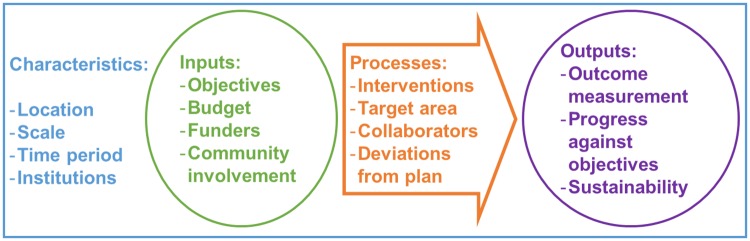
Framework used for describing the T&T control programmes.

For each document retained, relevant information which fell under the categories in Fig 2 was extracted and tabulated. When evaluating disease control programmes, process evaluation refers to the delivery and uptake of the programme and measures the extent to which the program has been implemented as planned. Outcome evaluation assesses the short and long-term impact of the programme and whether it has achieved its proposed goals [23]. The following were considered relevant outcome measures: changes in tsetse density, changes in disease frequency, changes in cattle productivity, and economic measures of the success of the control programme. The full tables are available as supporting information (S1–S5 Tables) and summary tables are presented in the following sections.

## Results

A total of 5,772 references were retrieved from the database search as well as additional sources (documents recommended by respondents during the interviews, from the grey literature or identified through reference lists). After removing duplicates (970), articles which could not be accessed (20) and non-relevant documents based on initial title and abstract screening (4,660), 122 documents were read in full to assess their eligibility. In total, 68 documents, describing 23 T&T control programmes, were eventually included in the review. A further 23 documents were used to contribute to the narrative of the situation in the five countries studied. The checklist and flow diagram of the literature review are provided as S1 and S2 Checklists. A total of twelve telephone interviews were carried out by AM. Ten respondents had experience and knowledge related to T&T control within the following countries: Burkina Faso (2), Cameroon (3), Ethiopia (1), Uganda (1) and Zambia (3), while two informants had multi-country expertise.

### T&T control in Burkina Faso

Three tsetse species are the primary vectors of bovine trypanosomiasis in Burkina Faso: *Glossina palpalis gambiensis* and *G*. *tachinoides* infest the riverine forests, whereas *G*. *morsitans submorsitans* subsists in some savannah areas [21, 24]. The northern limit of tsetse distribution in Burkina Faso is latitude 12°45’ and the strategy for the PATTEC campaign is to repel this limit gradually southwards–a so called “rolling carpet strategy” [25]. In the 1970-80s, droughts and demographic pressure in the semi-arid and arid areas in the Sahel regions led pastoralists to migrate southwards with their trypanosensitive cattle. This was coupled with livestock development in sub-humid fertile areas, including increasing use of crossbred animals. Farmers in these areas were subjected to high losses due to AAT [26]. Since then, several T&T control campaigns have been implemented in the country. Five well-documented control operations which were identified are presented in Table 1 (a detailed table for each country is available as supplementary data, S1 to S5 Tables).

**Table 1:** Most documented T&T control operations implemented in Burkina Faso since 1980.

Leading institutions	Location (scale)	Time period of project	Objectives	Level of community participation	Interventions	Reduction in tsetse level	Reduction in AAT level	Difficulties	Sustainability	Ref
CRTA	Sidéradougou (pastoral development zone 3,000 km^2^)	1983–1984	Tsetse elimination	Information	ITT + SIT	100%	92%	Delayed availability of funds. Occasional reintroduction through cattle migration	Unsustained. Barriers not maintained. Tsetse reinvasion via river networks	[24, 26–31]
CIRDES-CIRAD	Dafinso (20 km^2^)	1993–1995	Control of AAT epidemic	Project management. Financial contribution	ITT + ITC	100%	Not reported	Farmers could not meet the costs of control	Epidemics controlled. Return of AAT after end of campaign but perceived as less problematic	[32, 33]
PDRI-CIRDES	Padema (4,800 Km^2^)	1993–1999	Integrated T&T control	Project requested by the community. Project management. Financial contribution	ITT + ITC + TRY	95%	86%	Theft of traps. Low participation of community.	Unsustained. Tsetse reinvasion after end of campaign	[26, 33, 34]
CIRDES-ILRI	Yalé (pastoral development zone 400 km^2^)	1994–1997	Control of AAT epidemic	Resource contribution	ITT + ITC + TRY	98.4%	80%	Farmers could not meet the costs to continue control	Epidemics controlled. But tsetse reinvasion and return of AAT cases after end of campaign	[35–38]
PATTEC	“Boucle du Mouhoun” (40,000 km^2^)	2009 onward	Tsetse elimination	Labour contribution	ITT + ITC + SAS + ground spraying + TRY (SIT was planned)	99%	90%	Organisational issues	Sustained. Barriers in place	[26]

Abbreviations: CIRDES, Centre International de Recherche-Développement sur l’Élevage en zone Subhumide; CRTA, Centre de Recherche sur les Trypanosomoses Animales; ILRI, International Livestock Research Institute; ITC, insecticide-treated cattle; ITT, insecticide-treated traps and targets; PDRI, Projet de Développement Rural Intégré; SAS, sequential aerial spraying; SIT, sterile insect technique; TRY, trypanocidal drugs.

The longstanding involvement of the Centre de Recherche sur les Trypanosomoses Animales and later the Centre International de Recherche-Développement sur l’Élevage en zone Subhumide in T&T control in Burkina Faso has shaped the operations carried out in the last 35 years. The PATTEC and the Food and Agriculture Organisation are two of the other organisations heavily involved in T&T control in the country. Early research-oriented field experiments such as those in Sidéradougou and Yalé, designed as proofs of concept, were later followed by development-oriented projects. Despite attempts to engage with the community and involve them in control operations, these projects were not sustained after the withdrawal of research support and funds. One informant attributed these failures to two main factors, firstly, a loss of motivation to maintain control efforts once T&T levels are low and the disease is perceived as less important by the livestock owners. Public disengagement when control activities have reduced disease prevalence to low levels is a common pitfall of disease elimination campaigns and has been described in detail in public health [39]. The second incriminated factor relates to the control costs: as activities were initially provided free of charge to the beneficiaries, the transition to full cost recovery was difficult, preventing sustained control (researcher, Burkina Faso, September 2015).

The PATTEC-supported activities in Burkina Faso are currently financed by the government, following the end of funding from the African Development Bank. Recently they were re-oriented towards farmer-based control after failure to implement the elimination plans initially advocated (T&T control officer, Burkina Faso, August 2015). Currently, West Africa ecological characteristics are generally considered unsuitable for SIT, as genetic flows identified within the tsetse belt suggest that the sub-populations are not isolated [40–43]. Also, large areas in the region such as nature reserves tend to escape control operations for political, economic and environmental reasons, and act as tsetse reservoirs. However, recent studies based on genetic data [44, 45] suggest that small isolated tsetse populations exist on the edges of the tsetse belt and could be targeted by elimination campaigns, as is the case in the Niayes area in Senegal [46]. Although some of the informants engaged during this study believed that SIT is not an appropriate technique in the Burkinabe context due to ecological and logistical reasons, it is still on the agenda. The development of a tsetse factory near Bobo-Dioulasso was initiated under the PATTEC project and is continuing with financial support from the Ministry of Animal Resources (researcher, Burkina Faso, September 2015).

Long-term control seems extremely challenging in Burkina, and the veterinary services “can’t maintain targets for ever” (researcher, Burkina Faso, September 2015). Incorporating tsetse trapping and targets in community core activities is a possible alternative option for long term control. However, ITC seems to be more readily accepted by livestock farmers as part of their routine. “There is more motivation to apply insecticide treatments directly on livestock rather than in the environment and tick control is already routinely used. You just have to switch their usual acaricides for pyrethroids” (member of an international organisation, Burkina Faso, August 2015). Private veterinarians and veterinary drug dealers are key stakeholders in the process of privatising T&T control. Yet personal interests might bias their interventions, for instance by encouraging the use of trypanocidal drugs where they are not necessary. One informant stressed that the involvement of these actors in T&T control should be framed by guidelines to ensure the quality of their interventions (researcher, Burkina Faso, September 2015).

Trypanocides are currently the method favoured and most used by farmers to control AAT, because they are relatively accessible and their private benefits are perceived as high by livestock owners. They are widely and indiscriminately used in cattle-rearing areas; a study in Kénédougou estimated that trypanocides represent 42% of the veterinary drugs purchased by farmers, followed by anti-helminthic drugs [47]. The annual cost of importing trypanocides into the country is estimated to be 460 million FCFA (800,000 USD), accounting for legal imports only, it is estimated that the same amount may be imported through informal channels [48]. However, widespread and often improper use of trypanocides have led to increasing resistance, and since 2009 Burkina Faso has been part of a surveillance network for resistance to trypanocides and acaricides [49]. As an alternative to drugs, trypanotolerant taurine cattle (Baoulé and Lobi breeds) are also raised in Burkina Faso. Although larger Sahelian zebu breeds tend to be favoured for their higher productivity, they are highly susceptible to trypanosomiasis. Therefore the use of crossbred animals, which have some level of trypanotolerance whilst maintaining acceptable production levels, is increasing [26].

It is also important to note that the T&T situation in Burkina Faso is evolving under the pressure of agro-ecological factors. Due to the rarefaction of wildlife, the trypanosomiasis eco-epidemiological cycle becomes increasingly reliant on domestic hosts and thus easier to control [21]. Moreover, savannah tsetse have almost disappeared from Sahelian West Africa following the development of crop and cotton production and the increasing human pressure on the environment.

### T&T control in Cameroon

In Cameroon two thirds of the territory and 90% of the 6 million cattle are at risk of AAT. The Adamawa region is the main cattle rearing area in the country, supplying animal products to the national markets and neighbouring countries [50]. The plateau, which was first invaded by tsetse in 1950, hosts two species responsible for the transmission of AAT: *G*. *morsitans submorsitans*, and *G*. *tachinoides* [51]. Preventive and curative trypanocidal drugs were administered to cattle by government services from 1960 to 1975 in the regions of Adamawa, East and North. In the 1970s, control operations were reoriented towards vector control. They mainly targeted the Adamawa plateau, but DDT ground spraying operations were also conducted in the Far North region of Cameroon and adjacent regions of Nigeria [52]. The Mission Spéciale d’Eradication des Glossines (MSEG) was established in 1979 within the Ministry for Animal Production as a specialised tsetse unit incorporating existing vector control units. It was responsible for a long-term tsetse control program in the Adamawa region which ran from 1976 to 1994. The MSEG have maintained a technical and advisory role since 1994 but most of the T&T control in Adamawa is currently managed by the community itself. Details about the Adamawa campaign and the PATTEC campaign which is currently being planned are presented in Table 2.

**Table 2:** Most documented T&T control operations implemented in Cameroon since 1980.

Leading institutions	Location (scale)	Time period of project	Objectives	Level of community participation	Interventions	Reduction of tsetse level	Reduction of AAT prevalence	Difficulties	Sustainability	Ref
MSEG	Adamawa (35,000 km^2^)	1976–1994	Tsetse elimination	Information only. Farmers became involved in the protection of cleared areas after the end of the project	SAS + ITT + ITC	100% in core area (2005 study)	90% in core area (2005 study)	Repetitive reinvasion. Destruction of barriers by fire. Failure to transfer project to community	Sustained. Tsetse reinvasion prevented by ITC in buffer area. Core area still clear of tsetse.	[2, 52–56]
MSEG, PATTEC	Adamawa, North and Far North (AAT, 164,054 km^2^), South (HAT)	Not yet started.	AAT and HAT control	Not reported	ITT + ITC	Not applicable	Not applicable	Delays in obtaining funding. Tentative regional programmes aborted	Not applicable	[55, 57]

Abbreviations: ITC, insecticide-treated cattle; ITT, insecticide-treated traps and targets; MSEG, Mission Spéciale d’Eradication des Glossines; SAS, sequential aerial spraying.

Twenty years of vector control in Cameroon have had noticeable and lasting effects: a survey conducted in the 2000s showed that the Adamawa plateau was still cleared of tsetse. However, large areas in the Adamawa, North and Far North regions remain tsetse-infested and with high, and in some cases increasing, AAT prevalence levels [58]. A survey conducted in 2001 [56] revealed an AAT seroprevalence in cattle of 61% ± 8% in an infested zone of the Adamawa region. The lack of tsetse-free grazing areas in Cameroon to accommodate the growing cattle population and increasing conflicts in neighbouring Nigeria and Central African Republic forced some cattle owners to settle in the tsetse-infested areas of Cameroon, which were previously used only by nomadic herds. Over the last ten years, the number of cattle herds resident in the tsetse-infested area of Adamawa region jumped from 5 to 250 (researcher, Cameroon, November 2015). Insecticides and trypanocides are used on a continuous basis to prevent high mortality in these herds. The former contribute to maintaining the tsetse population at an acceptable level in the area. In spite of these measures, the mortality rates in cattle herds are relatively high and resistance of the trypanosomes to the drugs is widespread [59].

T&T control in Cameroon, as well as in other neighbouring countries, is now government-funded as the disease attracts less interest from international donors compared to other countries. Many expected that the launch of the PATTEC would be followed by increased external funding for T&T control and intense field activities but this has not happened in Cameroon to date (T&T control officer, Cameroon, September 2015). Therefore, T&T control is shared between the farmers and the MSEG, which is supported by sparse government resources, in the absence of external funding. Farmers are implementing ITC, ITT and trypanocides themselves and there is a real awareness among the communities of the problems caused by tsetse flies. However further technical assistance and coordination could improve the quality of farmer-based control. As an example, it is felt that a lot of the medicines (including trypanocides) purchased on the markets are of low quality, leading to treatment failures and increasing resistance (T&T control coordinator, Cameroon, October 2015). This has been supported by interviews with farmers in the region who mainly attributed treatment failure to fake or sub-standard drugs and cited “buying genuine drugs” as one of the main inconveniences with current AAT treatments [60]. Other control measures which need to be implemented at a higher level include the protection of national borders, for instance by buffer zones, to prevent the re-introduction of tsetse and trypanosomes in cleared areas. The intervention of the state is deemed essential by some informants: “the government must assist the farmers to make the control efforts efficient and sustainable” (researcher, Cameroon, November 2015). This appears to have been successfully achieved in the Faro and Deo district of the Adamawa region.

The tsetse population in this region of Africa covers several countries (Cameroon, Central African Republic, Chad, Congo, Gabon and Nigeria), therefore an integrated regional effort targeting whole tsetse belts irrespective of national borders is essential for sustained elimination in the area [61]. Despite 10 years of attempts to launch a regional control programme, all planned initiatives were aborted. Promoting regional coordination is a vital challenge for the sustainability of the control operations. These countries face similar challenges related to the ecological characteristics of the tsetse population and cattle transhumance practices. Twenty-four tsetse species are present in the region, from which at least seven (*G*. *fuscipes fuscipes*, *G*. *fuscipes quanzensis*, *G*. *morsitans submorsitans*, *G*. *pallicera pallicera*, *G*. *pallidipes*, *G*. *palpalis palpalis*, and *G*. *tachinoides*) are of epidemiological importance, compromising the use of techniques such as SIT. A large part of the region is occupied by largely inaccessible forests where only a limited range of control methods are usable and which serve as tsetse reinvasion sources (T&T control coordinator, Cameroon, October 2015). The tsetse-controlled zone in the Adamawa region is threatened by the proximity of two national parks (Gashaka in Nigeria and Faro in Cameroon) where tsetse thrive. In the absence of control in these parks, buffer zones could prevent reinvasion into the neighbouring grazing areas (researcher, Cameroon, November 2015).

Transhumance practices and international cattle movements are another serious obstacle to the control of T&T in the region. Border posts exist to treat cattle at their entrance into Cameroon (insecticides and trypanocides when necessary) but they cannot reach all transhumant herds entering the country. The MSEG encourages livestock farmers to adopt sedentary management, as it reduces the re-infestation problem posed by seasonal transhumance into infested areas. However, transhumance is deeply ingrained in these farmers’ cultural heritage and has been practiced for centuries. It is also a way of practicing livestock farming which is tailored to the ecological background of the region, making sustainable use of pastures and adapting to climatic events. To limit the consequences, the MSEG officers aim to treat the cattle leaving to and returning from infested areas with insecticides (T&T control officer, Cameroon, September 2015).

### T&T control in Ethiopia

The distribution of tsetse in Ethiopia covers up to 200,000 km^2^ in the South and West parts of the country [62]. Four species, *G*. *morsitans submorsitans*, *G*. *pallidipes*, *G*. *fuscipes fuscipes* and *G*. *tachinoides* are responsible for most AAT transmission in Ethiopia [63]. People and livestock tend to concentrate in the tsetse-free highlands, however there is increasing demographic pressure to expand agriculture into tsetse-infested areas. Tsetse belts expanded in the early 1970s to include areas such as the Didessa Valley, further restricting the areas suitable for livestock farming [64]. Countrywide chemotherapy operations started in cattle during the 1980s, but failed to control the disease which almost eliminated livestock in some areas, such as the Upper Didessa Valley [65]. Failure of chemotherapy control was partly attributed to wide-spread drug resistance in this area with some strains becoming resistant to all three drugs used for cattle treatment [66]. Therefore the government, assisted by international organisations, started to implement vector control campaigns in some areas. Sciarretta, Girma [62] reported that government spending on AAT was approximately 1 million USD per year. The government branch responsible for T&T control is the National Tsetse and Trypanosomiasis Investigation and Control Centre, which comes under the Ministry of Agriculture. Besides government-lead operations, some non-governmental organisations, such as FARM-Africa, and research institutes, such as the International Livestock Research Institute (ILRI), also conducted T&T control campaigns in Ethiopia. The most documented campaigns conducted in Ethiopia since 1980 are presented in Table 3.

**Table 3:** Most documented T&T control operations implemented in Ethiopia since 1980.

Leading institutions	Location (scale)	Time period of project	Objectives	Level of community participation	Interventions	Reduction of tsetse level	Reduction of AAT prevalence	Difficulties	Sustainability	Ref
NTTICC	Upper Didessa Valley (4,500 km^2^)	1986–1989	Vector control	Labour contribution	ITT	100% *G*. *m*. *submorsitans* (*G*. *tachinoides* remained at low levels)	91%	Not reported	Sustained. No GMS reinvasion. Barriers in place. Control of GT in the 2000s. Current status not documented	[14, 64, 65, 67]
ILCA	Ghibe River Valley (150 km^2^)	1990–1992	Vector control	Not reported	ITT + TRY (+ ITC later)	74 to 81% depending on species	85%	Thefts of traps. Socio-political disturbance	Unsustained. Tsetse reinvasion. Second phase from 1993, mixed results	[68, 69]
ILCA	Ghibe River Valley (200 km^2^)	1991–1993	Vector control	Resources contribution. Information. Farmers’ groups	ITC + TRY	0 to 93% depending on species	60%	Slow decline in tsetse densities	Sustained. Control still in place 5 years later. Full cost recovery scheme.	[66, 70–72]
FARM-Africa	Konso (350 km^2^)	1995–2000	AAT control	Training. Resources contribution	ITC + TRY	90%	100%	Low level of coverage, explained by treatment fee and lack of information	Unsustained. Possible tsetse reinvasion.	[73]
ICIPE	Luke (50 km^2^)	1995–2004	Integrated disease control	Initial request. Resources contribution	ITT + TRY	80%	66%	Not reported	Sustained. Project progressively handed over to community. Current status not documented	[62, 74, 75]
STEP / PATTEC	Southern Rift Valley (25,000 km^2^)	1997 onwards	Tsetse elimination	Training. Labour contribution.	ITT + ITC + TRY + SAS + ground spraying + SIT	Up to 95%	Up to 90%	Delays in SIT. Logistic and project management issues	Sustained. Barriers in place.	[76–81]
EIAR	Metekel	2011–2012	Vector and AAT control	Training of 66 community animal health workers for target management	ITT + TRY	84%	70%	Not reported	Not reported	[82]

Abbreviations: EIAR, Ethiopian Institute of Agricultural Research; ICIPE, International Centre of Insect Physiology and Ecology; ILCA, International Livestock Centre for Africa; ITC, insecticide-treated cattle; ITT, insecticide-treated traps and targets; NTTICC, National Tsetse and Trypanosomiasis Investigation and Control Centre; SAS, sequential aerial spraying; SIT, sterile insect technique; STEP, Southern Ethiopia Tsetse Eradication Project; TRY, trypanocidal drugs.

Ethiopia provides several examples of sustainable T&T control programmes. This allows us to describe particular circumstances and contexts which seem to maximize the chances than an intervention is sustainable. Government-led operations initiated at the end of the 1980s in the Upper Didessa Valley have been sustained for years, leading to improved cattle health and production levels in the area. Changes in land use following tsetse control, such as an increase in the amount of land under crop cultivation, prevented tsetse flies from re-invading the cleared areas by rendering the environment unsuitable. Two rare examples of successful community-based programs have also been described in the country. The Luke community scheme highlights the advantages of integrating T&T control into a wider rural development project, improving the likelihood of long-term benefits of the activities. The ILRI project in the Ghibe Valley was handed over to the community after a cost-recovery scheme had been introduced in the second year of the project [71]. Through our literature search and interviews we have not been able to identify field data that would allow an assessment of the current situation in the target areas and the impact of these initiatives up to the present day. Although the outcomes and impacts of past programmes would be a key element in the planning and design of new control activities, these data are rarely available in the area of T&T. The small number of studies reviewed during the course of this work does not reflect the large number of programmes which have been trialled and implemented in Ethiopia, some of which are still ongoing. The majority of these initiatives have been either initiated or supported by the government, which plays a significant role in assisting farmers with T&T control efforts in this country. There is abundant information available on T&T distribution in various regions of Ethiopia, however a large part of it consists of grey literature which might not be readily available to researchers and control implementers in other countries. Moreover, a significant amount of these data are outdated as the distribution of tsetse flies and trypanosomes is evolving with time (T&T researcher, Italy, February 2016).

Last, it is difficult to estimate the proportion of AAT cases due to mechanical transmission in tsetse-infested areas where both the cyclical and the mechanical routes are involved. An informant (T&T control officer, Ethiopia, September 2015), as well as a study [82], reported that *Trypanosoma vivax* (which can be transmitted by both types of flies, tsetse and biting flies) tends to become more prevalent than other trypanosome species in areas where tsetse flies are controlled. This suggests that the mechanical transmission of AAT is non-negligible in this country and this should not be neglected within T&T control activities [83].

### T&T control in Uganda

Uganda is heavily affected by AAT and both Gambian and Rhodesian forms of HAT. Four species of flies are epidemiologically important in the country: *G*. *fuscipes fuscipes*, *G*. *morsitans submorsitans*, *G*. *pallipides* and *G*. *brevipalpis*. The two latter species were eliminated in some areas in the 1980s, following drastic reduction in their wildlife hosts. *G*. *fuscipes fuscipes* is an opportunistic feeder and more resilient to the anthropization of its habitat [84]. However, re-invasion by *G*. *pallipides* into several districts has been reported since the end of the 1990s, increasing the risk of AAT in livestock [85]. Half of the cattle population are at risk of AAT, which is endemic in most Ugandan districts, except some highland areas such as the South-West [86]. Numerous control operations have been reported and the most documented are described in Table 4.

**Table 4:** Most documented T&T control operations implemented in Uganda since 1980.

Leading institutions	Location (scale)	Time period of project	Objectives	Level of community participation	Interventions	Reduction of tsetse level	Reduction of AAT prevalence	Difficulties	Sustainability	Ref
Government services	Busoga (2,200 km^2^)	1988–1990	Control of HAT epidemic	Labour contribution	ITT + ITC	99%	100% reduction of HAT in certain parishes	Persistent tsetse reinvasion from adjacent areas	Not reported. However similar HAT control was sustainable in neighbouring Tororo	[87–89]
LHRI	Busia (130 km^2^)	1991–1993	AAT control	Not reported	ITC	98.4%	92%	Diminution of coverage led to disease upsurge	Not reported	[90, 91]
LHRI	Tororo (1,350 km^2^)	1991–1995	AAT and HAT control	Information only	ITT +/- ITC +/- TRY	99.5%	94%	Persistence of mechanical transmission	Lack of funding but AAT prevalence still lower in control area in 2000	[92, 93]
FITCA	Busoga (2,000 km^2^)	1999–2004	AAT and HAT control	Resource contribution. Farmers’ groups to manage ITC. Community assistants for ITT	ITT + ITC + zero-grazing units	75 to 90%	‘Insufficient’	Low level of coverage. Farmers’ groups did not persist in time.	Unsustained. Tsetse reinvasion by 2009	[14, 94–97]
PATTEC	South-East (15,000 km^2^)	2005	Tsetse elimination	Information only. Stronger involvement was planned	ITC + TRY (SIT planned)	50 to 75% in 12,000 km^2^	Not reported	Failure to implement SIT. Failure to reorient project due to management issues.	Unsustained. Funding came to an end in 2011. Tsetse reinvasion may occur in the absence of barriers	[98–101]
Makerere University	North-East (8,800 km^2^)	2006	Control of HAT epidemic	Resources contribution	ITC + TRY	Not reported	75%. Large reduction of HAT prevalence	Low level of coverage due to community hesitance. Wide use of amitraz. Cattle immigration.	Unsustained. The control activities were not followed by community involvement.	[94, 102–105]

Abbreviations: FITCA, Farming In Tsetse Controlled Areas project; ITC, insecticide-treated cattle; ITT, insecticide-treated traps and targets; LHRI, Livestock Health Research Institute; SIT, sterile insect technique; TRY, trypanocidal drugs.

AAT and HAT control are inter-dependent in Eastern Uganda, where cattle are a reservoir for the trypanosomes responsible for Rhodesian HAT, with a prevalence of *Trypanosoma brucei* (subspecies *T*. *brucei brucei* and *T*. *brucei rhodesiense*) of up to 40% among some Ugandan cattle populations [106]. Currently both AAT and Rhodesian HAT are endemic in the South-East of the country, while Gambian HAT is endemic in the North-West of the country, although the distribution of the different trypanosome species is shifting. Movement of cattle from endemic areas of South-East Uganda to Eastern and more Northerly districts has introduced the disease into areas previously free of the zoonotic species where tsetse were already present, resulting in sleeping sickness epidemics in humans [107, 108]. The last campaign described in Table 4, a public-private partnership control campaign entitled Stamping Out Sleeping Sickness, was launched in 2006 in order to stop *T*. *brucei rhodesiense* from spreading further north, which could result in a dramatic overlap of the Gambian and Rhodesian forms of sleeping sickness [102]. As tsetse were invading new areas of Uganda during the 20^th^ century, numerous control campaigns have been conducted in various areas of the country but none have been sustainable in the long-term [84]. Following the failure of top-down approaches, several studies were conducted on the feasibility of community-based interventions. These programmes, such as the FITCA and Stamping Out Sleeping Sickness campaigns, faced sustainability issues as well. Community-based projects faced management and financial issues, which may be attributed to a lack of engagement of the project recipients. As a consequence, the levels of coverage achieved using ITT and/or ITC were insufficient to interrupt the transmission of the parasites. Tsetse trapping is usually regarded as the responsibility of the government, although farmers are often willing to contribute labour to their deployment and maintenance. However, they are usually willing to pay for treatment of their own cattle with insecticides and trypanocides as the benefits of these techniques are perceived as private [109]. Another major limiter of T&T control operations in Uganda is the political unrest up to the early 1990s. Although early T&T control campaigns were initially successful, conflicts often disturbed the operations, discontinuing them completely in some areas [2, 110]. More recently, efforts to control tsetse in the North-West of the country using so-called “tiny targets” yielded promising results, and the method was deemed to be an effective tool for HAT control [111].

### T&T control in Zambia

Approximatively a third of Zambia’s territory is composed of wildlife estates: National Parks where neither residency nor hunting is permitted, and large Game Management Areas, where residency is permitted and hunting is regulated through the attribution of hunting licenses. Tsetse flies are thriving with an abundance of hosts in these conservation areas and the Luangwa and Zambezi valleys are thought to have the highest density of tsetse in Africa [112]. Four tsetse species are of epidemiological importance in Zambia: *G*. *morsitans morsitans*, *G*. *morsitans centralis*, *G*. *pallidipes* and *G*. *brevipalpis* [113]. Table 5 presents the well-documented control operations implemented in Zambia since 1980. During the past 50 years, the Tsetse Control Services conducted numerous SAS campaigns, covering large areas mainly in the Western Province [16, 114]. However, there is a lack of published data on the characteristics and achievements of these activities. The control activities in the Kwando-Zambezi area, which aimed at the elimination of this entire tsetse belt, are continuing under the guidance of the PATTEC since 2008. In the East of the country, a number of SAS operations as well as ITT campaigns have been run under the RTTCP umbrella. As re-invasion of most of the tsetse-free areas occurred, owing to the absence or lack of efficiency of barriers, elimination was not reached by the end of this program in 1995 [115].

**Table 5:** Most documented T&T control operations implemented in Zambia since 1980.

Leading institutions	Location (scale)	Time period of project	Objectives	Level of community participation	Interventions	Reduction of tsetse level	Reduction of AAT prevalence	Difficulties	Sustainability	Ref
Government services	West province (2,000 km^2^)	1987–1989	Tsetse elimination	Labour contribution. Long-term involvement was planned.	ITT + TRY	100%	93%	Failure to hand over management of the traps to the community	Sustained. Barriers in place. Extension of control to 11,500 km^2^	[116–118]
RTTCP	East province (900 km^2^)	1989–1994	Tsetse control	No	SAS + ITT	100%	82%	Low density of targets	Sustained in core area only. Barriers in place. Current status not documented	[115, 119, 120]
PATTEC	Kwando-Zambezi belt (22,000 km^2^)	2008 onwards	Tsetse elimination	Paid labour contribution. Information	SAS + ITT	100%	100%	Organisational issues. Slow progress	Sustained. Barriers in place.	[121]

Abbreviations: ITT, insecticide-treated traps and targets; RTTCP, Regional Tsetse and Trypanosomiasis Control Programme; SAS, sequential aerial spraying; TRY, trypanocidal drugs.

The Zambian case provides another example of control operations with impacts that have been sustainable in time. Several factors may explain the success of control operations in Zambia in terms of sustainability, when compared to other countries, including ecological, political and economic factors. First the tsetse ecological characteristics are more favourable for elimination as populations are isolated within three main tsetse belts: Kwando-Zambezi, Lower Zambezi and Eastern (also referred to as ‘Common’) belts. Second, effective and sustained regional cooperation enabled the four countries (Angola, Botswana, Namibia and Zambia) involved in the Kwando-Zambezi Control Project to make enormous progress in the elimination of the eponym belt. Third, the Zambian government is entirely supporting the control efforts, which makes the availability of external funding less critical for the long-term viability of the control. However the T&T situation is far from being under control. Endemic equilibrium with low mortality prevails in a number of areas but movements of cattle closer to the nature reserves regularly lead to epidemic phases [6]. Farmers and their livestock migrate closer and closer to the wildlife conservation areas due to population growth and increasing natural constraints such as droughts, therefore increasing their exposure to tsetse. Despite existing coping strategies, such as keeping only a couple of trypanocide-treated oxen to work tsetse-infested land while the rest of the herd graze in low-risk areas, AAT cases (as well as HAT cases) are regularly reported in these human-wildlife interface areas (T&T control officer, Zambia, February 2016).

An informant underlined that AAT is not an attractive disease for decision-makers (T&T control officer, Zambia, February 2016). This is partly due to the chronic nature of the disease, which is neither a critical issue for international trade (such as foot-and-mouth disease) nor a disease causing visible and pathognomonic symptoms (such as anthrax). Moreover, the absence of up-to-date entomological and parasitological data at national level as well as data on economic impact on households is a disadvantage for T&T control projects when they are submitted to potential funders. Among veterinary services as well, AAT is not a high-priority disease. “AAT is there but it’s not in the focus [of the veterinary services], it is number 4 or 5 in terms of the diseases that they attend to, East Coast fever, contagious bovine pleuro-pneumonia and foot-and-mouth disease are the top-three currently” (researcher, Zambia, February 2016). Despite this, when farmers in tsetse-infested areas of the Eastern Province of Zambia were asked to list important livestock diseases, AAT was the most commonly listed disease in one study area and second in another, after lumpy skin disease [122]. In addition, the most affected areas are often remote with poor infrastructure, including limited access to veterinary services, which is likely to result in under-reporting and unawareness of the true impact of disease by decision makers (researcher, England, January 2016). As a consequence, most of the disease control is implemented by the farmers themselves, who use preventive and curative trypanocides. However, a large proportion of farmers have only limited access to drugs because of the remoteness of their communities (researcher, Zambia, February 2016).

Local community-based projects emerged in Zambia in the late 1990s, aiming at T&T suppression rather than elimination. According to an informant, the communities are eager to benefit from control operations as they are most aware of the impact of the disease on their households (researcher, Zambia, January 2016). But despite this initial enthusiasm, the few community-based projects documented faced sustainability issues. This may be partly due to failure of the projects to achieve a strong engagement of farmers in the project activities. For example, farmers in a cleared area of the Western Province were eager to participate in the maintenance of traps, however, they did not know how to perform this and wanted more training on the control of AAT (researcher, England, January 2016). During a study conducted in the Eastern Province most farmers in one study area were unsuccessful at identifying pictures of tsetse traps. They stated that they had seen them previously but had not known their purpose and that traps are often taken down to use the material for other purposes [122]. Another study reported that lack of an institutional framework to assist farmers implementing vector control methods such as ITC in the longer term may explain the failure of the programmes to be sustainable [123].

## Discussion

Data on previous T&T control programmes implemented in similar contexts are extremely useful in the design phase of new interventions. Yet the results of this review demonstrate that T&T control projects are rarely submitted to a full evaluation, similarly to many other development projects. Despite the availability of guidelines and frameworks for the assessment of disease control programmes [23, 124, 125], unbiased evidence of effectiveness, socio-economic and environmental impacts and sustainability of the operations were usually not demonstrated. Further, although documentation on the inputs, processes and outputs of the interventions included in this review might be available within the implementing institutions, the data available in the public domain were limited. Documentation of economic aspects was even scarcer, only one of the articles identified included a full economic evaluation and was conducted in the 1980’s [116]. Economic evaluations are more common for HAT control programmes (see a review by Sutherland, Yukich [126]).

Standardised evaluation of T&T control programmes is needed for two main reasons. Firstly, such evaluations, acknowledging the successes and failures of the interventions, would increase the accountability of the programmes, which have been entrusted with billions of US dollars of public and private funds. Secondly, the documentation resulting from the monitoring and evaluation of T&T control interventions should be summarised and made accessible in the public domain in order to be exploited as a resource for decision making regarding future control interventions. Such data would provide donors and policy makers with evidence on the most appropriate options for control, within the local epidemiological, social and environmental contexts. The experience gained from past control programmes would allow potential problems to be identified before they arise. Eventually, decision makers also need measures of the benefits arising from their control programmes. This information is required for priority setting, in order to decide whether AAT control is a worthwhile investment compared to other diseases, in which settings the interventions are likely to be successful and sustainable and which methods appear to be the most cost-beneficial. Several publications studied the economic benefits of T&T control but did not relate them to the costs of the control programme [38, 67]. Ultimately the economic returns of control programmes should be calculated in relation to their costs allowing cost-benefits of programmes using different strategies and in different settings to inform future resource allocation. A number of costing studies and benefit cost-analyses have been conducted but were mostly based on the simulation models of hypothetical scenarios [46, 99, 100, 127–129], except for a cost analysis carried out on an AAT control project in south-eastern Uganda, which was based on real costs [11]. In addition, there can be much variability in the inputs of economic analysis in terms of costs included and new revenue considered. An overall benefit cost ratio higher than one was cited as a prerequisite for a successful human disease eradication programme [130] and it is likely to be important for animal diseases as well. There is a need for standardised, simple frameworks and decision-making tools to allow planners to rank areas for AAT control, compare costings of different strategies and aid decision making regarding which strategies to implement. In addition, the likely distribution of the benefits for different stakeholders should be estimated, particularly for integrated HAT/AAT control programmes, in order to generate evidence to encourage appropriate individuals and groups to take responsibility for particular costs. Project planning should always include an assessment of technical feasibility, based on available resources, existing infrastructure and the ecological and epidemiological context as well as an assessment of the likely sustainability of the control activities, as it will assist the tailoring of the project to the local context. These preliminary assessments might require an extensive data collection phase when such data are not available. For example, informants from Zambia and Cameroon reported a lack of up-to-date information concerning the actual distribution of tsetse populations and trypanosome burden in these countries. Historical data are usually available but might be out-of-date as tsetse distributions are continuously affected by progressive anthropization of the environment as well as climate change.

Overall, only a limited number of peer-reviewed publications describing T&T control programmes was available, creating the need to collect information from other sources such as grey literature and key informants to complete the description of the interventions presented in this study. The validity of expert opinion is often criticised, but in some cases it can be the only available source of information. The outputs of expert elicitation should not be disconnected from the process via which they have been obtained, as the opinions of informants reflect their own perceptions of the interventions in which they have been involved. In spite of the limited amount of data available, key themes which influenced the success or failure of the selected control programmes became apparent from the available literature. These elements are described in the following paragraphs.

Most of the techniques, or combinations of techniques, used within the programmes described above reduced tsetse densities and AAT prevalence with measurable effects within a few months. However, sustainability, defined as their long-term viability [33], was reportedly a central issue for most of the control programmes identified in this review. Most control operations target non-isolated tsetse populations, leading to a permanent re-invasion pressure on tsetse-cleared areas. For instance, genetic studies showed that tsetse flies from adjacent river basins within Burkina Faso and those of neighbouring countries (Mouhoun, Sissili, Comoé and Niger River basins) regularly migrate between those areas and control zones [42]. Moreover, 7% of the PATTEC target area in Burkina Faso is covered by protected natural areas which are currently not dealt with and threaten the cleared areas [26]. Therefore, barriers to reinvasion need to be continually maintained to protect cleared areas whilst tsetse are still present in neighbouring areas. Besides reinvasion, resurgence of the tsetse population in areas deemed to have been cleared is also common, as tsetse are able to recover from very low levels of density which are not detectable by conventional sampling methods [105, 131]. Ideally, control operations should not cease until a complete isolated population of tsetse has been eliminated. This requires long-term efforts and commitment from all the actors involved in control, which is hardly compatible with the very definition of a “project”. The target area and the timescale to be covered by a project are usually subject to available funding, resources or even research aims, whereas for sustainability purposes they should ideally be defined to suit the biological characteristics of the target organism as well as other non-biological factors. For instance, the potential conflicts between the objectives set by international institutions or donors and the needs of the recipient communities have been highlighted by Enserink [78] in relation to the role of the International Atomic Energy Agency in the promotion of SIT. Ideally, local stakeholders (from government authorities to farmers, traditional authorities, and agricultural extension services) should be involved in the early planning stages, but they are often consulted at a later stage, when crucial decisions about the type, scale and characteristics of the proposed intervention have already been taken. To ensure sustainability, T&T control interventions should aim at the highest levels of “interactive participation” and “self-mobilization” of the typology described by Pretty [132]. This is not the case for most of the interventions reviewed here, with the notable exception of the rural development project in the Luke community, Ethiopia, which arose from a request of the community itself [75]. In the context of T&T control projects aiming to transfer responsibility of control at the end of a project, it is crucial that the recipients are both willing and able to assume this. A few projects such as the ILRI activities in the Ghibe Valley initiated in 1991 were supported by the recipient community for at least a few years following the end of the external funding, but no data were available on their viability in the longer term. No evidence of successful projects sustained entirely by the recipient community in the long term were found during the preparation of this review. The long-term participation of communities in T&T control operations has been linked to three key elements [26, 33, 133, 134]: the costs of control should be supported by the beneficiaries, a strong technical support should be provided to the community actors and the motivation of all actors should be sustained in time. Farmers are more likely to adhere to and financially support control operations when they believe that the private benefits of these control activities are higher than the public benefits. For instance, insecticide and trypanocide treatments for cattle are both a private and public good as the reduction in AAT and HAT levels may benefit the whole community. Cattle owners are usually willing to pay for these treatments for their animals as the perceived private benefits are high. Other measures such as ITT are much less attractive to farmers, as their benefits are considered to be mainly public. There are successful examples of incorporation of cost recovery schemes within control projects, such as the scheme in the Ghibe Valley in Ethiopia, although this may lead to a drop in participation when the benefits are not perceived as being worth the costs. The provision of technical support, the second element cited above, to enable communities to effectively implement the selected control measures and to transfer the necessary management skills, is frequently neglected, leading to the projects’ collapse [33]. Both livestock extension services and local non-governmental organisations may play a key role in such training and knowledge transfer activities [135]. Last, the sustained motivation of all actors is also critical. As mentioned by most of the informants, the initial motivation of the farmers to participate in control programmes is usually high, especially during epidemic phases when mortality is significant within their herds. A study in Uganda showed that the factors contributing to the acceptance were the knowledge of symptoms and transmission of African Trypanosomiasis, the perception of the disease risk and coexistence with tsetse, supernatural beliefs, perception of the efficiency of traps in past control operations and willingness to be involved in tsetse control operations [136]. Some types of farmers, such as nomadic farmers, may be more difficult to involve in T&T control operations, as they tend to be less involved in community life than their sedentary counterparts [137]. Some community-based projects such as Community Animal Health Workers initiatives in East Africa were largely successful in the long-term. One of their advantages over T&T control is that they are based on broad animal-health interventions [138]. Some T&T control activities (e.g. FITCA and Luke community in Ethiopia, Padéma in Burkina Faso) are integrated into wider rural development initiatives but this is the exception, not the rule. The benefits of such interventions are still perceptible once the AAT incidence has been reduced to negligible levels. Broad animal health interventions may be more appropriate to meet the priorities and expectations of the recipients in the longer term, which is fundamental for sustained motivation and cooperation. For the same reason, ITC and insecticide-treated nets are techniques usually well accepted by livestock owners, as they protect their cattle against a range of nuisances which may include tsetse, ticks, biting flies and mosquitoes. It is also more likely to be sustained once the tsetse population has been reduced below detectable levels, as the other benefits of the treatments remain.

Decision-making about the appropriate level of governance and funding is complex in areas where tsetse belts stretch over several countries or where cross-border trade and movements of trypanosensitive livestock exist (often due to transhumance or the political situation). As tsetse are highly mobile, regional level coordination is necessary in these cases but might be hampered by political disagreement or by differences between the countries’ own agendas. The Kwando-Zambezi Control Project stands as a successful example of regional project, whereas other attempts such as a regional control programme within the Communauté Économique et Monétaire de l’Afrique Centrale were aborted due to lack of commitment from some of the partners. It is critical that international institutions such as PATTEC foster such partnerships, as they did in the case of the Kwando-Zambezi Control Project. The appropriate level of governance is also linked to the choice of techniques, some of them requiring institutional or governmental leadership as they need to be applied on relatively large scale, such as SAS and SIT. Other techniques are more appropriate for farmer- or community-based control activities such as ITC and use of trypanocidal drugs. The increase in the range of available control techniques has always been accompanied by vehement debates on their respective values and potential uses. This culminated with PATTEC’s statement that it intended to make use of the sterile insect technique. This revived the debate on when and where this technique would be suitable within the scientific and decision-making communities. This also relates to the debate on whether elimination or control of tsetse should be the goal of the campaigns. Some advocate that the use of SIT to control T&T in mainland Africa is unrealistic and unaffordable and that locally-targeted control of the disease should be set as a goal in most areas [7, 139–142]. Others argue that control is not sustainable nor cost-effective in the long-term. Some of those support the use of SIT to eliminate remaining tsetse after an initial reduction phase [2, 18, 105, 143, 144]. Although the use of SIT was initially planned on a large scale in three countries (Burkina Faso, Uganda and Ethiopia) within the Phase I of the PATTEC, only the Deme Valley in Ethiopia is being treated so far. Two main obstacles explain these delays. First no insect rearing facility is currently in a position to produce the weekly amount of sterilized males that would be necessary to implement SIT on a larger surface and second, SIT should be applied on a previously suppressed and naturally or artificially isolated tsetse population, which has not yet been achieved in the other study areas. Sustainable elimination of tsetse using SIT has only been described on the island of Unguja, Zanzibar [145], but a number of programmes led to the eradication of other pests such as screwworm in North and Central America for example [146].

Whether T&T control falls under public or private responsibility is another unresolved debate as T&T control creates both public (for instance on public health where HAT is also present and on food security) and private benefits (for instance on household income from livestock production) [135]. Control of the vector creates positive externalities for all farmers in the target area, not just those actively involved in control e.g. using ITC, which may justify some public (or external) investment. Examples of public, private and mixed funding of the T&T control have been detailed in this review. The Kwando-Zambezi project is an example of entirely government-funded and -implemented programme which has proved successful and sustainable so far, due to a long-term substantial commitment of the relevant services. As opposed to the ephemeral nature of “projects”, government-based T&T control units provide a durable structure which guarantee some continuity for the control activities. Nonetheless such examples are rare since structural adjustment plans have left the veterinary services of many African countries with very limited personnel and financial resources available. Where national resources are limited, external sources of funding (e.g. international aid, non-governmental organisations, and philanthropists) are a major support to control projects, with the sustainability issues mentioned above. A significant number of initiatives are mixed public-private partnerships, based on an initial financial input from the donor followed by provision of the recurrent costs from the community, such as some of the current PATTEC activities in Burkina Faso. Some authors suggested that free market laws alone cannot support the T&T control efforts, as they would favour the use of cheaper acaricides instead of tsetse effective products. Moreover, key subpopulations which may threaten the overall reduction of T&T, such as transhumant livestock, may not be covered because treatment is not cost-effective for those farmers. Therefore the implementation of a policy framework based on market restriction allowing only tsetse effective products as well as compulsory treatment of migrating animals has been proposed [104]. Such a close collaboration between public institutions and private actors could be a key element to ensure the control is well anchored within the socioeconomical context and sustainable. This may negate the need to establish a governmental department in charge of T&T control, if one does not already exist, and reduce administrative costs. Long-term T&T control or elimination is an ambitious enterprise. The different elements listed above advocate for mixed public-private partnerships which would be more likely to be sustained in time. A close integration between disease elimination activities and the broader human and animal health care services was deemed crucial to the success of the eradication campaigns for smallpox and rinderpest respectively [147, 148]. It is also cited as a key factor for the elimination of HAT [149, 150]. Political and societal factors also have a major impact on the outcome of disease elimination programmes, as it has been reported for malaria and poliomyelitis for example [39, 151, 152]. However, the evolution of such factors is difficult to predict, especially when the disease impact becomes less visible and public support wanes.

## Conclusions

The numerous T&T control operations implemented over the last century provide a wealth of information in terms of the efficiency and costs of the techniques available to date, as well as the successes and failures of different logistical, financial and institutional arrangements. Yet the results of this review conducted over five countries show that evaluations of these control programmes using standardised methodologies are rare and that the dissemination of the information related to the interventions is limited. International initiatives such as PATTEC could play a crucial role in encouraging the use of standardised evaluation protocols. Sharing the knowledge gained from past interventions outside the implementing institutions and countries is crucial to sustain the continental dynamic initiated by the PATTEC towards the eradication of tsetse. The sector will only gain from strengthening communication and data sharing with other sectors, and thriving towards integration of T&T control with the broader human and animal health care services.

## Supporting Information

S1 Checklist PRISMA checklist of the literature review. (DOCX)

S2 Checklist Flow diagram of the literature review. (TIF)

S1 Table Detailed description of five well-documented control operations implemented in Burkina Faso since 1980. (DOCX)

S2 Table Detailed description of two well-documented control operations implemented in Cameroon since 1980. (DOCX)

S3 Table Detailed description of seven well-documented control operations implemented in Ethiopia since 1980. (DOCX)

S4 Table Detailed description of six well-documented control operations implemented in Uganda since 1980. (DOCX)

S5 Table Detailed description of three well-documented control operations implemented in Zambia since 1980. (DOCX)
