# Supplementary figures and images for: Past and Ongoing Tsetse and Animal Trypanosomiasis Control Operations in Five African Countries: A Systematic Review
Source: PLoS Negl Trop Dis. 2016 Dec 27;10(12):e0005247. doi: 10.1371/journal.pntd.0005247 (PMC5222520; doi:10.1371/journal.pntd.0005247)

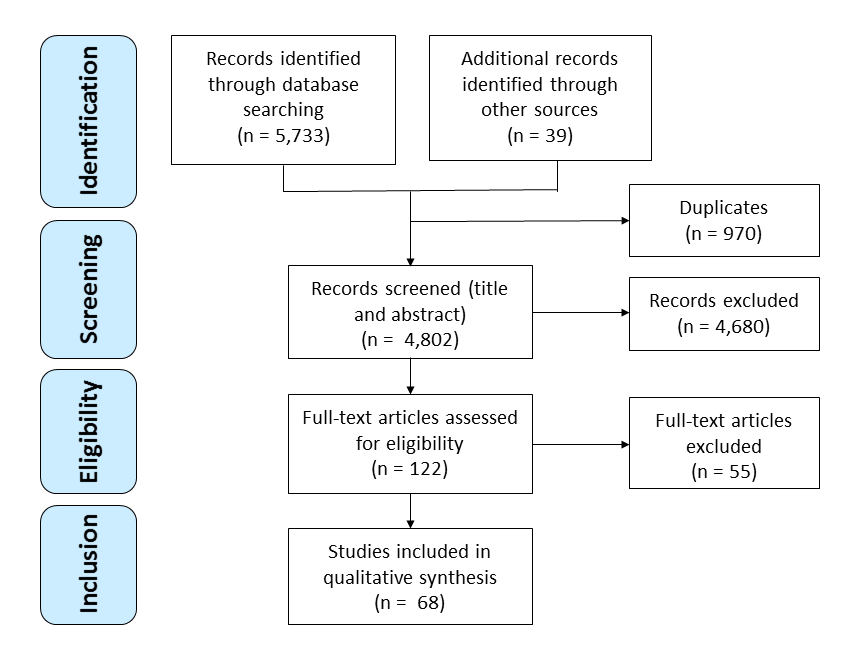

Supplement: S2 Checklist — (TIF) [file pntd.0005247.s002.tif]
