# Supplementary material for: Past and Ongoing Tsetse and Animal Trypanosomiasis Control Operations in Five African Countries: A Systematic Review
Source: PLoS Negl Trop Dis. 2016 Dec 27;10(12):e0005247. doi: 10.1371/journal.pntd.0005247 (PMC5222520; doi:10.1371/journal.pntd.0005247)
Supplement: S1 Table — (DOCX) [file pntd.0005247.s003.docx]

S1 Table. Detailed description of five well-documented control operations implemented in Burkina Faso since 1980

Abbreviations:

- General: AAT, Animal African Trypanosomiasis; CIRAD, Centre International de Recherche Pour l’Agriculture et le Développement; CIRDES, Centre International de Recherche-Développement sur l’Élevage en zone Subhumide; CRTA, Centre de Recherche sur les Trypanosomiases Animales; HAT, Human African Trypanosomiasis; ILRI, International Livestock Research Institute; PDRI/HKM, Project de Dévelopement Rural Intégré / Houet-Kossi-Mouhoun; T&T, tsetse and trypanosomiasis
- Tsetse species: GMS, *Glossina morsitans submorsitans*; GPG, *Glossina palpalis gambiense*; GT, *Glossina tachinoides*
- Interventions: DA, diminazene aceturate; DLT, deltamethrin; GS, ground spraying; ISM, isometamidium salt; ITC, insecticide-treated cattle; ITT, insecticide-impregnated traps and/or targets; SIT, sterile insect technique; TRY, use of trypanocidal drugs

| **Project** | **CRTA work, 1983-84, Sidéradougou** |
| --- | --- |
| **Objectives** | Tsetse elimination |
| **Interventions** | - ITT: 7200 DLT-impregnated screens on 600 km river galleries in dry season - SIT, releases during 2 rainy seasons - Barriers of targets |
| **Location** | Pastoral Development Zone of Sidéradougou in the Comoé province |
| **Surface of target area** | 3,000 km^2^ |
| **Initial target population** | 70,000 cattle |
| **Tsetse species** | *GT, GPG, GMS* |
| **Trypanosome species** | *T. congolense, T. vivax, T. brucei* |
| **Budget** | Not available |
| **Funders** | French cooperation and German international aid agency |
| **Year starting** | 1983 |
| **Duration of project** | 3 years |
| **Collaborators & implementers** | Centre de Recherche sur les Trypanosomoses Animales (now CIRDES) and Ecole de Lutte Anti-Tsétsé |
| **Involvement of community** | Information only. |
| **Deviations, set-backs and difficulties** | Some minor reinvasion occurred in the North through migration of cattle but was controlled by trapping. Delays due to financial and technical issues. |
| **Outcome measurement** | Elimination of riverine species reached after 2 years. No fly caught in 1985. Reduction of population of *GMS* to a lesser extent. 92% reduction of AAT level. |
| **Progress against the objectives** | Elimination reportedly achieved. Bouyer, Seck [1] suggest that the elimination may not have been achieved because sensitivity of the trapping was low. |
| **Sustainability** | Barriers were removed shortly after they were handed over to government services, tsetse flies re-invaded the area via the river networks as shown by a survey in 1986. GMS did not re-establish due to anthropogenic alterations of the ecosystem. |
| **References** | [1-8] |

| **Project** | **PDRI/HKM – CIRDES work, 1993-99, Padéma** |
| --- | --- |
| **Objectives** | Community-based control programme to:   - overcome repetitive failures to sustain elimination - overcome tensions between different types of farmers due to competition over resources (water, land) |
| **Interventions** | - ITT: 1500 screens, impregnated every 2 months - ITC: DLT spray every 2 months - TRY: DA in infected cattle |
| **Location** | Padema district in the Mouhoun River Basin |
| **Surface of target area** | ~ 4,800 km^2^ (27 villages) |
| **Initial target population** | 10,000 cattle |
| **Tsetse species** | *GT*, *GPG*, *GMS* |
| **Trypanosome species** | *T. congolense, T. vivax, T. brucei* |
| **Budget** | The project’s main objective was to limit public investment and make farmers responsible for the project. |
| **Funders** | Food and Agriculture Organisation (Programme de Lutte contre la Trypanosomose) |
| **Year starting** | 1993 |
| **Duration of project** | 6 years |
| **Collaborators & implementers** | PDRI/HKM, CIRDES, and private veterinarians |
| **Involvement of community** | The community contributed to the implementation and maintenance of treated targets through labour and farmers partially covered the costs of the cattle treatments (insecticides and trypanocides). The remainder of the costs were covered by the project. |
| **Deviations, set-backs and difficulties** | Two thirds of the traps were destroyed (flooding) after 6 months, and participation in cattle treatments continually declined, leading to the campaign being suspended in 1995. Farmers did not meet the requested financial contribution. |
| **Outcome measurement** | 86% reduction in AAT levels was achieved with a 95% reduction of tsetse. |
| **Progress against the objectives** | Success |
| **Sustainability** | Project not sustainable. Possible reasons for failure were:   - Technical constraints (import of fabric for targets, gathering of farmers on pulverisation days) - Poor management of the project and its finances by the village committees not used to these processes - Social context: transhumant cattle owners excluded from the project because of non-recognition of their importance by the locals - Tensions and lack of cooperation between private (vets, tailors) and public actors (animal health officers) - Failure of the project team to recognize and deal with these issues in time.   Discussions with the actors involved led to a second phase of control in 1996-99, in Padéma and adjacent Solenzo. Although experience had been gained from the first phase, full participation and a transfer of responsibility from the PDRI/HKM to the community has not been achieved. By 1999, farmers reported the return of tsetse and high losses due to AAT. |
| **References** | [6, 9, 10] |

| **Project** | **CIRAD - CIRDES work, 1993-1995, Dafinso** |
| --- | --- |
| **Objectives** | Control ongoing AAT epidemic through integrated disease management |
| **Interventions** | - ITT: 60 conic traps - ITC: DLT 5% pour-on monthly |
| **Location** | Dafinso village in the Houet province |
| **Surface of target area** | 20 km^2^ (1 village) |
| **Initial target population** | 300 cattle |
| **Tsetse species** | *GT* mainly, *GPG* |
| **Trypanosome species** | *T. congolense, T. vivax, T. brucei* |
| **Budget** | Not available |
| **Funders** | French cooperation |
| **Year starting** | 1993 |
| **Duration of project** | 3 years |
| **Collaborators & implementers** | CIRAD, CIRDES |
| **Involvement of community** | Programme was based on an initial request from the community, who were heavily reliant on treatment of cattle. Community paid for the traps and the pour-on. A village association was formed. |
| **Deviations, set-backs and difficulties** | Failure of farmers to pay back the initial loan and the recurrent costs. |
| **Outcome measurement** | Tsetse under detection threshold by June 1994. Reduction of AAT level. |
| **Progress against the objectives** | Success |
| **Sustainability** | Not sustainable because most farmers were unable to support costs. Return of T&T at lower levels than before the campaign. |
| **References** | [10, 11] |

| **Project (ctd.)** | **CIRDES-ILRI work 1994-1997, Yalé** |
| --- | --- |
| **Objectives** | Control ongoing AAT epidemic (since 1989) through integrated disease management |
| **Interventions** | - ITC: 6-18/km^2^ DLT 1% pour-on every other month in rainy season - ITT: 1500 screens along river in dry season - TRY: DA in infected cattle |
| **Location** | Pastoral Development Zone of Yalé in the Sissili province |
| **Surface of target area** | 400 km^2^ |
| **Initial target population** | 1,500 cattle |
| **Tsetse species** | *GT, GMS* |
| **Trypanosome species** | *T. vivax, T. congolense* |
| **Budget** | Not available |
| **Funders** | French cooperation, European Union |
| **Year starting** | 1994 |
| **Duration of project** | 4 years |
| **Collaborators & implementers** | Ministry of Agriculture, CIRDES, ILRI, and private veterinarians |
| **Involvement of community** | The community contributed labour in the form of trap maintenance and money to the purchase of trypanocides. |
| **Deviations, set-backs and difficulties** | The introduction of a treatment fee lead to a decrease in ITC coverage. |
| **Outcome measurement** | 98.4% reduction in tsetse population, no more infected flies were detected until 1997. 89% reduction in cattle annual mortality. 80% reduction in AAT prevalence in cattle. |
| **Progress against the objectives** | Success (tsetse density under detection threshold in the core area) |
| **Sustainability** | The traps were removed at the end of the project, since the livestock owners’ contribution was not enough to maintain this system. Therefore, tsetse densities increased again after 1997, due to the presence of highly infested areas nearby. |
| **References** | [6, 12-15] |

| **Project (ctd.)** | **Current PATTEC campaign** |
| --- | --- |
| **Objectives** | Tsetse elimination |
| **Interventions** | - ITT: 40,000 impregnated screens along the rivers during dry season - ITC was planned for the rainy season but delayed because of organisational issues, however, cattle were treated with epicutaneous cypermethrin during the trypanocidal treatment campaign - SAS and GS conducted in certain areas - TRY: DA mass treatment, associated with preventive ISM in high-risk areas - Barriers (ITT + GS) on both extremities of the treated river section |
| **Location** | “Boucle du Mouhoun” region |
| **Surface of target area** | 40,000 km^2^ |
| **Initial target population** | 2 million cattle |
| **Tsetse species** | *GPG, GT* |
| **Trypanosome species** | *T. vivax, T. congolense* |
| **Budget** | 14 million USD (Phase I) (= 350 USD/km^2^) |
| **Funders** | African Development Fund (phase I) |
| **Year starting** | November 2009 |
| **Duration of project** | Phase I funding ceased in 2013, current control costs now supported by the government |
| **Collaborators & implementers** | T&T Control Unit within the Department for Veterinary Services |
| **Involvement of community** | Communities provided labour for the installation of the traps and screens and were responsible for their maintenance, but all costs were borne by the project. |
| **Deviations, set-backs and difficulties** | Persistence of AAT transmission during rainy seasons could be due to mechanical transmission of *T.vivax*. ITC and ground spraying delayed due to organisational issues. |
| **Outcome measurement** | Reduction (over 99%) in tsetse populations after 6 months. 90% reduction in the incidence of animal AAT was achieved in sentinel herds. |
| **Progress against the objectives** | Elimination not achieved |
| **Sustainability** | Tsetse kept under control until present, using two barriers of traps and targets. The project plans to use SIT to eliminate the tsetse fly in the intervention area, which is the only way to stop the current control measures. An insectarium is under construction. Studies show that reinvasion can occur from adjacent river basins in Burkina Faso and neighbouring countries and protected natural areas, that have not been cleared yet [6, 16]. Therefore barriers will have to be maintained to prevent reinvasion from neighbouring areas, until they are cleared as well. |
| **References** | [6, 16] |

**References**

1. Bouyer J, Seck MT, Sall B. Misleading guidance for decision making on tsetse eradication: Response to Shaw et al. (2013). Prev Vet Med. 2013;112(3–4):443-6.

2. Cuisance D, Politzar H, Merot P, Tamboura I. Irradiated Male Release for an Integrated Campaign against Glossina Spp in the Sideradougou Pastoral Area (Burkina). Rev Elev Med Vet Pays Trop. 1984;37(4):449-67.

3. Politzar H, Cuisance D. An Integrated Campaign against Riverine Tsetse, Glossina-Palpalis-Gambiensis and Glossina-Tachinoides, by Trapping, and the Release of Sterile Males. Insect Sci Appl. 1984;5(5):439-42.

4. Merot P, Politzar H, Tamboura I, Cuisance D. Results of a Control Campaign against River Tsetse Flies in Burkina Using Deltamethrine Impregnated Screens. Rev Elev Med Vet Pays Trop. 1984;37(2):175-84.

5. Cuisance D, Politzar H, Tamboura I, Mérot P. Coût de l'emploi de barrières de pièges et d'écrans insecticides pour la protection de la zone pastorale d'accueil de Sidéradougou, Burkina Faso. Rev Elev Med Vet Pays Trop. 1990;43(2):207-17.

6. Sow A, Sidibé I, Bengaly Z, Bouyer J, Bauer B, Van den Bossche P. Fifty years of research and fight against tsetse flies and animal trypanosomosis in Burkina Faso. An overview. Bull Anim Health Prod Afr. 2010;58(2):95-118.

7. Bauer B, Petrich-Bauer J, Kabore I, Kourouma B, Mattausch M, Some J, et al. Epidemiological survey in the pastoral zone of Sideradougou, Burkina Faso. Modern Insect Control: Nuclear Techniques and Biotechnology, Vienna (IAEA-SM, 301/44). 1988:139-49.

8. De La Rocque S, Augusseau X, Guillobez S, Michel V, De Wispelaere G, Bauer B, et al. The changing distribution of two riverine tsetse flies over 15 years in an increasingly cultivated area of Burkina Faso. Bulletin of entomological research. 2001;91(03):157-66.

9. Bontoulougou J, Oule J-M, Pellissier J-P, Tallet B. La participation des acteurs, un exercice difficile: Leçons de l'expérience d'un plan de lutte contre la trypanosomose animale africaine dans la vallée du Mouhoun (Burkina Faso). Nat Sci Soc. 2000;8(1):33-43.

10. Grace D. Participative trypanomosis control in Burkina Faso, lessons learned, ways forward. Report from the International Research Institute for Livestock, Nairobi, Kenya; 2003.

11. Amsler-Delafosse S, Kabore I, Bauer B. Lutte contre les vecteurs de la trypanosomose animale africaine au Burkina Faso. Cah Agric. 1995;4(6):440-3.

12. Bauer B, Amsler-Delafosse S, Kabore I, Kamuanga M. Improvement of cattle productivity through rapid alleviation of African animal trypanosomosis by integrated disease management practices in the agropastoral zone of Yale, Burkina Faso. Trop Anim Health Pro. 1999;31(2):89-102.

13. Kamuanga M, Swallow BM, Sigué H, Bauer B. Evaluating contingent and actual contributions to a local public good: Tsetse control in the Yale agro-pastoral zone, Burkina Faso. Ecol Econ. 2001;39(1):115-30.

14. Kamuanga M, Sigue H, Swallow B, Bauer B, d'Ieteren G. Farmers' perceptions of the impacts of tsetse and trypanosomosis control on livestock production: evidence from southern Burkina Faso. Trop Anim Health Pro. 2001;33(2):141-53.

15. Kamuanga M, Kabore I. La lutte contre les glossines dans la zone agropastorale de Yalé (Burkina Faso): résultats d’enquêtes socio-économiques. Tropicultura. 2005;23(3):146-53.

16. Kone N, Bouyer J, Ravel S, Vreysen MJ, Domagni KT, Causse S, et al. Contrasting population structures of two vectors of African trypanosomoses in Burkina Faso: consequences for control. PLoS Negl Trop Dis. 2011;5(6):e1217.
