# Supplementary material for: Past and Ongoing Tsetse and Animal Trypanosomiasis Control Operations in Five African Countries: A Systematic Review
Source: PLoS Negl Trop Dis. 2016 Dec 27;10(12):e0005247. doi: 10.1371/journal.pntd.0005247 (PMC5222520; doi:10.1371/journal.pntd.0005247)
Supplement: S2 Table — (DOCX) [file pntd.0005247.s004.docx]

**S2 Table. Detailed description of two well-documented control operations implemented in Cameroon since 1980**

Abbreviations:

- General: AAT, Animal African Trypanosomiasis; HAT, Human African Trypanosomiasis; MSEG, Mission Spéciale d’Éradication des Glossines; T&T, tsetse and trypanosomiasis
- Tsetse species: GMS, *Glossina morsitans submorsitans*; GFF, *Glossina fuscipes fuscipes*; GT, *Glossina tachinoides*
- Interventions: ITC, insecticide-treated cattle; ITT, insecticide-impregnated traps and/or targets

| **Project** | **Adamawa, main control area** |
| --- | --- |
| **Objectives** | Tsetse elimination |
| **Interventions** | - Aerial spraying by helicopter in dry seasons (dieldrin, endosulfan, deltamethrin, cypermethrin) - Barriers: ITT and ITC |
| **Location** | Adamawa plateau |
| **Surface of target area** | 35,000 km^2^ (core area= 25,000 km^2^) |
| **Initial target population** | Not available |
| **Tsetse species** | *GMS*, *GFF* and *GT* depending on districts |
| **Trypanosome species** | *T. congolense* mainly, *T. vivax*, *T. brucei* |
| **Budget** | 30 million USD over 10 years to treat 21,000 km^2^ (1,400 USD/km^2^) |
| **Funders** | Food and Agriculture Organisation, World Bank and Cameroonian government |
| **Year starting** | 1976 |
| **Duration of project** | 18 years (operations disrupted in the 1980s and suspended in 1994) |
| **Collaborators & implementers** | MSEG |
| **Involvement of community** | Information only. Some training and incentives to use ITC were provided in very limited areas. At the end of the aerial spraying campaigns in 1994, farmers became the principal actors of T&T control in Adamawa, using mainly ITC. |
| **Deviations, set-backs and difficulties** | Reinvasion of the plateau from the North side occurred several times. A barrier of traps and targets was set to protect the plateau but were soon destroyed by bush fire. A buffer zone were ITC was implemented was created.  The contract between the MSEG and the World Bank stipulated that the funding of the vector control should have been gradually transferred to the community, as well as most of its execution. Failure to inform and train the project recipients led to the funding coming to an end in 1994. |
| **Outcome measurement** | By the end of the control activities in 1994, the core area had been cleared of tsetse and AAT was under control. Study in 2004-05 showed that the plateau was still clear of tsetse and AAT reduced by 90 % compared to its level in the valley. |
| **Progress against the objectives** | Elimination achieved in the core area |
| **Sustainability** | Following the interruption of the spraying campaigns in 1994, limited reinvasion occurred from the neighbouring areas. Surveys conducted in the 2000s showed that the plateau (core area) was still cleared of tsetse but *GMS* and *GT* were caught in the buffer zone and the valley. AAT prevalence is lower on the plateau compared to the valley and the buffer zone, showing that the earlier T&T operations have had lasting effects. However, tsetse reinvasion and transhumance may lead to the re-establishment of the disease in the whole area. |
| **References** | [1-6] |

| **Project** | **Planned campaign** |
| --- | --- |
| **Objectives** | Control of AAT and HAT |
| **Interventions** | - ITT - ITC, especially during transhumance when cattle graze in heavily infested areas |
| **Location** | Adamawa plateau, North and Far North  (and South Cameroon for HAT control) |
| **Surface of target area** | 164,054 km^2^ |
| **Initial target population** | 4 million cattle (Adamawa only) |
| **Tsetse species** | *GMS*, *GFF* and *GT* depending on districts |
| **Trypanosome species** | *T. congolense*, *T. brucei*  *(T. vivax* is rare, mostly imported) |
| **Budget** | Tentative budget 1 to 1.2 million USD |
| **Funders** | Government |
| **Year starting** | Not started yet |
| **Duration of project** | Not applicable |
| **Collaborators & implementers** | MSEG, with assistance from PATTEC |
| **Involvement of community** | Not applicable |
| **Deviations, set-backs and difficulties** | Delays in obtaining funding. Several regional programmes (with Chad, Central African Republic and Nigeria) have been proposed but aborted. |
| **Outcome measurement** | Not applicable |
| **Progress against the objectives** | Not applicable |
| **Sustainability** | Not applicable |
| **References** | [4, 7] |

**References**

1. Mueller P, Nagel P, Flacke W. Ecological side effects of Dieldrin application against tsetse flies in Adamaoua, Cameroon. Oecologia (Berlin). 1981;50(2):187-94.

2. Mamoudou A, Zoli A, Mbahin N, Tanenbe C, Bourdanne, Clausen PH, et al. Prevalence and incidence of bovine trypanosomosis on the Adamaoua plateau in Cameroon 10 years after the tsetse eradication campaign. Vet Parasitol. 2006;142(1-2):16-22.

3. Mamoudou A, Zoli A, Tchoua P. Parasitological prevalence of bovine trypanosomosis in the Faro and Deo division valley of the Adamaoua plateau, Cameroon. International Journal of Biological and Chemical Sciences. 2009;3(5):1192-7.

4. Mamoudou A, Zoli A, Van den Bossche P, Delespaux V, Cuisance D, Geerts S. Half a Century of Tsetse and Animal Trypanosomosis Control on the Adamawa Plateau in Cameroon. Revue d'Élevage et de Médecine Vétérinaire des Pays Tropicaux. 2009;62(1):33-8.

5. Tanenbe C, Gambo H, Musongong G, Boris O, Achukwi M. Prévalence de la trypanosomose bovine dans les départements du Faro et Déo, et de la Vina au Cameroun: bilan de vingt années de lutte contre les glossines. Revue d'élevage et de médecine vétérinaire des pays tropicaux. 2010;63(3-4):63-9.

6. Allsopp R. Options for vector control against trypanosomiasis in Africa. Trends in Parasitology. 2001;17(1):15-9.

7. Banipe L. Pan African Tsetse and Trypanosomiasis Eradication Campaign - Exposé du Cameroun. 11ème Réunion des Coordonnateurs Nationaux/Points Focaux PATTEC; 10-11 December 2012; Hawassa, Ethiopia2012.
