# Supplementary material for: Past and Ongoing Tsetse and Animal Trypanosomiasis Control Operations in Five African Countries: A Systematic Review
Source: PLoS Negl Trop Dis. 2016 Dec 27;10(12):e0005247. doi: 10.1371/journal.pntd.0005247 (PMC5222520; doi:10.1371/journal.pntd.0005247)
Supplement: S3 Table — (DOCX) [file pntd.0005247.s005.docx]

**S3 Table. Detailed description of seven well-documented control operations implemented in Ethiopia since 1980**

Abbreviations:

- General: AAT, Animal African Trypanosomiasis; FAO, Food and Agriculture Organisation; ILCA, International Livestock Centre for Africa; NTTICC, National Tsetse and Trypanosomiasis Investigation and Control Centre and community; STEP, Southern Tsetse Eradication Project; T&T, tsetse and trypanosomiasis
- Tsetse species: GMS, *Glossina morsitans submorsitans*; GP, *Glossina pallipides*; GT, *Glossina tachinoides*; GFF, *Glossina fuscipes fuscipes*
- Interventions: DA, diminazene aceturate; DLT, deltamethrin; ITC, insecticide-treated cattle; ITT, insecticide-impregnated traps and/or targets; SAS, sequential aerial spraying; SIT, sterile insect technique; TRY, use of trypanocidal drugs

| **Project** | **NTTICC work, 1986-89, upper Didessa Valley** |
| --- | --- |
| **Objectives** | Vector control campaign to manage AAT in spite of the drug resistance problem |
| **Interventions** | - ITT: DLT-impregnated traps and odour-baited screens |
| **Location** | Limu Shay, Chelo, Debelle and Dembi-Toba villages in the upper Didessa Valley, Oromia State |
| **Surface of target area** | 4,500 km^2^ |
| **Initial target population** | Not reported |
| **Tsetse species** | *GMS*, *GT* |
| **Trypanosome species** | *T. congolense, T. vivax* |
| **Budget** | Not reported |
| **Funders** | FAO, Ethiopian government |
| **Year starting** | 1986 |
| **Duration of project** | 3 years |
| **Collaborators & implementers** | NTTICC |
| **Involvement of community** | Members of the community provided the labour for the deployment and maintenance of the traps and targets under the supervision of tsetse control officers. |
| **Deviations, set-backs and difficulties** | Not reported |
| **Outcome measurement** | Over 91% reduction in AAT incidence. Increase in cattle health and productivity parameters. *GMS* disappeared from the area while *GT* remained at low levels. |
| **Progress against the objectives** | Success |
| **Sustainability** | Barriers prevented reinvasion of the area. By 1996, elimination activities had stopped and an area of 4,500 km^2^ in the Upper Didessa Valley was considered tsetse-cleared. A study in 2004 showed that the effects of the control campaign were still sustained (cattle mortality 73% lower in tsetse-controlled areas) and had led to an improvement of livestock production and crop-livestock integration. Between 1999 and 2004, ITT and ITC were implemented within another control programme to control *GT*. |
| **References** | [1-4] |

| **Project** | **ILCA work, 1990-92, Ghibe Valley** |
| --- | --- |
| **Objectives** | Vector control campaign to manage AAT in spite of the drug resistance problem |
| **Interventions** | - ITT: DLT-impregnated screens, 5/km^2^ - TRY: DA in infected cattle |
| **Location** | Medale site in the Ghibe River Valley, SNNPR State |
| **Surface of target area** | 150 km^2^ |
| **Initial target population** | Not reported |
| **Tsetse species** | *GFF*, *GP* |
| **Trypanosome species** | *T. congolense, T. vivax* |
| **Budget** | Not reported |
| **Funders** | ILCA, FAO and other funders |
| **Year starting** | 1990 |
| **Duration of project** | 3 years |
| **Collaborators & implementers** | ILCA |
| **Involvement of community** | Not reported |
| **Deviations, set-backs and difficulties** | Repeated thefts of the traps and targets associated with socio-political disturbances in Ethiopia. |
| **Outcome measurement** | 74% reduction in *GFF* density (resp. 81% of *GP*) in the first months but returned to pre-control levels within a year. Initial 60% reduction in AAT but rose to pre-campaign level by 1992. |
| **Progress against the objectives** | Not achieved |
| **Sustainability** | The operations were disrupted due to socio-political circumstances. Following the invasion of the area by *GMS* and the increasing impact of AAT, another control operation was conducted from December 1993 to 1998 with monthly deltamethrin pour-on treatments. Reduction in AAT incidence was observed, but effective reduction of tsetse population was achieved only in areas frequented by cattle. Alongside the river, the tsetse population was not affected, probably due to high reinvasion pressure and low contact between tsetse and treated cattle. |
| **References** | [5, 6] |

| **Project** | **ILCA work, 1991-93, Ghibe Valley** |
| --- | --- |
| **Objectives** | Vector control campaign to manage AAT |
| **Interventions** | - ITC: 10-20/km^2^ cypermethrin monthly pour-on - TRY: DA in infected cattle |
| **Location** | Tolley/Gullele site in the Ghibe River Valley, SNNPR State |
| **Surface of target area** | 200 km^2^ |
| **Initial target population** | 4,000 cattle |
| **Tsetse species** | *GFF*, *GP*, *GMS* |
| **Trypanosome species** | *T. congolense, T. vivax* |
| **Budget** | Not reported |
| **Funders** | ILCA, FAO and other funders |
| **Year starting** | 1991 |
| **Duration of project** | 3 years |
| **Collaborators & implementers** | ILCA and International Laboratory for Research on Animal Diseases |
| **Involvement of community** | Treatment fee introduced in 1992. Information campaigns and knowledge surveys in the area. Farmers’ groups responsible for control operations after first year. |
| **Deviations, set-backs and difficulties** | Cypermethrin seems to have a shorter persistence on cattle than deltamethrin, leading to slower declines in the tsetse levels |
| **Outcome measurement** | No reduction in *GFF* density, 83% reduction in *GMS* density and 93% reduction in *GP* density. 85% reduction in AAT prevalence. Reduction in other problems caused by biting flies and protected cattle from ticks. Increase in the efficiency of animal traction and reduction in abortion rates and calf mortality. |
| **Progress against the objectives** | Success |
| **Sustainability** | Barriers were kept in place. Farmers in the control area perceived the benefits generated by the treatment to be high and were willing to pay for it. The introduction of the full cost-recovery scheme in December 1992 did not hamper the reduction in tsetse populations, and the number of animals treated in 1993 was greater than the previous year. However, the difficulty to access rural areas in Ethiopia and the ability of farmers to pay market prices for the drugs would probably constrain delivery of the treatment by the private sector. A paper reported that the control scheme was still ongoing in 1996, under the cost recovery scheme. |
| **References** | [7-10] |

| **Project** | **FARM-Africa work, 1995-2000, Konso** |
| --- | --- |
| **Objectives** | Community-based AAT control |
| **Interventions** | - ITC: DLT pour-on, 18/km^2^ - TRY: DA in infected cattle |
| **Location** | Konso in SNNPR State |
| **Surface of target area** | 300-400 km^2^ (5 parishes) |
| **Initial target population** | 5,500 cattle |
| **Tsetse species** | Not reported |
| **Trypanosome species** | *T. vivax, T. congolense* |
| **Budget** | Not reported |
| **Funders** | Department For International Development (UK) |
| **Year starting** | 1995 |
| **Duration of project** | 5 years |
| **Collaborators & implementers** | FARM-Africa organisation |
| **Involvement of community** | Training and information were provided to the community. Farmers contribute through labour and fees (the latter were introduced in the 2^nd^ year). |
| **Deviations, set-backs and difficulties** | Very low coverage was achieved after the 2^nd^ year. |
| **Outcome measurement** | 100% reduction in AAT prevalence, 90% reduction in tsetse density |
| **Progress against the objectives** | Success |
| **Sustainability** | Costs of the treatment and lack of information were blamed for the low coverage after the 1^st^ year. All operations had ceased by 2000. |
| **References** | [11] |

| **Project** | **ICIPE work, 1995 onwards, Luke community** |
| --- | --- |
| **Objectives** | Integrated disease control through adaptive ecosystem management |
| **Interventions** | - ITT: odour-baited traps - TRY: treatment of infected animals |
| **Location** | Luke community in the SNNPR State |
| **Surface of target area** | 50 km^2^ |
| **Initial target population** | Not reported |
| **Tsetse species** | *GP*, *GMS* |
| **Trypanosome species** | *T. congolense, T. vivax, T.brucei* rare |
| **Budget** | Not reported |
| **Funders** | World Bank, through the Ethiopia Social Rehabilitation and Development Fund |
| **Year starting** | 1995 |
| **Duration of project** | Funded until 2004 |
| **Collaborators & implementers** | International Centre of Insect Physiology and Ecology (Nairobi) and community |
| **Involvement of community** | Request for assistance arose from the community itself. The community was increasingly involved in the project, both in tsetse monitoring and trap management, with the target of handing the project over to the community from 2005 onwards. |
| **Deviations, set-backs and difficulties** | Not reported |
| **Outcome measurement** | 80% reduction in tsetse density. 66% reduction in AAT prevalence. Over 10 years of control, large improvements in cattle production were achieved: 10-fold increase in cattle numbers, increase in calving rate, milk production and surface of cultivated land and reduction in calf mortality. This initiative also brought positive social changes. |
| **Progress against the objectives** | Success |
| **Sustainability** | From 2004, the existing scheme was adapted to improve its cost-effectiveness, while maintaining the low tsetse and AAT levels achieved in the previous phase.  Self-organization in the community will be needed for this program to be sustained and other issues arising to be managed. |
| **References** | [12-14] |

| **Project** | **Current PATTEC campaign** |
| --- | --- |
| **Objectives** | Tsetse elimination (following suppression phase) |
| **Interventions** | - ITT - ITC: pour-on - SAS and ground spraying - TRY: treatment of infected animal - SIT |
| **Location** | Southern Rift Valley (tsetse population more or less isolated by surrounding mountains) |
| **Surface of target area** | 25,000 km^2^ (block 1: 10,500 km^2^) |
| **Initial target population** | ~ 2 million cattle |
| **Tsetse species** | 5 species present. Block 1: GP and GFF only. |
| **Trypanosome species** | *T. congolense* and *T. vivax* mainly*, T.brucei* |
| **Budget** | 14 million USD (= 560 USD/km^2^) |
| **Funders** | African Development Fund |
| **Year starting** | STEP initiated in 1997 but funding from Phase I of PATTEC only awarded in 2007 |
| **Duration of project** | 7 years (phase I) |
| **Collaborators & implementers** | STEP, joint project of the Ethiopian Government and the International Atomic Energy Agency |
| **Involvement of community** | Training, information campaign, labour contribution. Strong involvement in suppression phase and responsibility for management of barriers were initially planned. |
| **Deviations, set-backs and difficulties** | Tsetse fly factory built in 2007. Major delays in the project implementation due to delays in the procurement of the gamma cell irradiator, which was set up in 2014 only. The initial delays have also been attributed to logistics and project management issues. |
| **Outcome measurement** | Lower mortality and higher fertility are reported in cattle from areas under control in the Districts of Arba Minch and Kindo-Koysha. A study in the Wolaita zone, within the STEP project, achieved 90% reduction of AAT in 2 areas with respectively ITT and ITC.  Within the PATTEC target area, tsetse populations were successfully suppressed (up to 95%) and SIT is currently underway in the Deme Valley (7% of the project area). A buffer zone of 35,000 km^2^ has also been created, with 65 to 90% reduction achieved in that area. Improvement in livestock health and production has been achieved. |
| **Progress against the objectives** | Not achieved |
| **Sustainability** | The SIT-phase of the elimination is underway. The factory is also expected to supply Kenya and Uganda elimination projects with irradiated flies. |
| **References** | [15-20] |

| **Project** | **Ethiopian Institute of Agricultural Research, 2011-2012, Metekel** |
| --- | --- |
| **Objectives** | Community-based vector control |
| **Interventions** | - ITT: 1,010 DLT-impregnated targets - TRY: DA in infected cattle |
| **Location** | Metekel zone in Benshangul Gumuz State |
| **Surface of target area** | ~ 10,700 km^2^ (22 villages within this area) |
| **Initial target population** | 77,000 cattle |
| **Tsetse species** | *GT* |
| **Trypanosome species** | *T. congolense, T. brucei, T. vivax* |
| **Budget** | Not reported |
| **Funders** | Ethiopian Institute of Agricultural Research, Canadian Physicians for Aid and Relief |
| **Year starting** | 2011 |
| **Duration of project** | 1 year |
| **Collaborators & implementers** | Ethiopian Institute of Agricultural Research, Pawe Agricultural Research Center |
| **Involvement of community** | Information of the community on T&T impact and control. Training of 66 community animal health workers for target management. |
| **Deviations, set-backs and difficulties** | Reduction in AAT prevalence lower than reduction in vector density, possibly due to the effect of mechanical transmission. |
| **Outcome measurement** | 84% reduction in tsetse density, 70% reduction in AAT prevalence |
| **Progress against the objectives** | Success |
| **Sustainability** | Not reported |
| **References** | [21] |

**References**

1. Jemal A, Hugh-Jones ME. Association of tsetse control with health and productivity of cattle in the Didessa Valley, western Ethiopia. Prev Vet Med. 1995;22(1–2):29-40.

2. Slingenbergh J. Tsetse control and agricultural development in Ethiopia. World Animal Review. 1992;70/71:30-6.

3. Regassa F, Abebe G. The Socio-economic Impact of Tsetse Control: the Case of Upper Didessa Valley, Western Ethiopia. Ethiop Vet J. 2009;13(1):81-103.

4. Agriconsortium. End of project evaluation study for FITCA regional and national components of five countries, Kenya, Uganda, Tanzania, Rwanda and Ethiopia. Report from the Farming In Tsetse Controlled Areas project, 2005.

5. Leak SG, Peregrine AS, Mulatu W, Rowlands GJ, D'Ieteren G. Use of insecticide-impregnated targets for the control of tsetse flies (Glossina spp.) and trypanosomiasis occurring in cattle in an area of south-west Ethiopia with a high prevalence of drug-resistant trypanosomes. Trop Med Int Health. 1996;1(5):599-609.

6. Rowlands GJ, Leak SGA, Peregrine AS, Nagda SM, Mulatu W, d'Ieteren GDM. The incidence of new and the prevalence and persistence of recurrent trypanosome infections in cattle in southwest Ethiopia exposed to a high challenge with drug-resistant parasites. Acta Trop. 2001;79(2):149-63.

7. Leak SGA, Mulatu W, Rowlands GJ, Dieteren GDM. A trial of a cypermethrin pour-on insecticide to control Glossina pallidipes, G. fuscipes fuscipes and G. morsitans submorsitans in south-west Ethiopia. Bull Entomol Res. 1995;85(2):241-51.

8. Swallow BM, Mulatu W, Leak SGA. Potential demand for a mixed public-private animal health input: evaluation of a pour-on insecticide for controlling tsetse-transmitted trypanosomiasis in Ethiopia. Prev Vet Med. 1995;24(4):265-75.

9. Omamo SW, Kagwanja J, Reid R, Ieteren G, Ndiwa N, Nyabenge M, et al. Agricultural extension reform in Africa: Insights and lessons from livestock disease control in South-West Ethiopia. Report from the International Livestock Research Institute, Nairobi, Kenya; 2002.

10. Rowlands GJ, Mulatu W, Leak SG, Nagda SM, d'Ieteren GD. Estimating the effects of tsetse control on livestock productivity-a case study in southwest Ethiopia. Trop Anim Health Pro. 1999;31(5):279-94.

11. Pound B, Jonfa E. Cattle in Southern Ethiopia: Participatory studies in Wolaita and Konso woredas. Report from the FARM-Africa, 2006.

12. Sciarretta A, Girma M, Tikubet G, Belayehun L, Ballo S, Baumgartner J. Development of an adaptive tsetse population management scheme for the Luke community, Ethiopia. J Med Entomol. 2005;42(6):1006-19.

13. Baumgärtner J, Gilioli G, Tikubet G, Gutierrez AP. Eco-social analysis of an East African agro-pastoral system: Management of tsetse and bovine trypanosomiasis. Ecol Econ. 2008;65(1):125-35.

14. Baumgärtner J, Tikubet G. From Tsetse Control to Sustainable Rural Development-Progress and Opportunities for an Ethiopian Community. In: Adisa R, editor. Rural Development - Contemporary Issues and Practices: InTech; 2012.

15. Gechere G, Terefe G, Belihu K. Impact of tsetse and trypanosomiasis control on cattle herd composition and calf growth and mortality at Arbaminch District (Southern Rift Valley, Ethiopia). Trop Anim Health Pro. 2012;44(7):1745-50.

16. Taye M, Belihu K, Bekana M, Sheferaw D. Assessment of impacts of tsetse and trypanosomosis control measures on cattle herd composition and performance in southern region, Ethiopia. Trop Anim Health Pro. 2012;44(7):1759-63.

17. Enserink M. Welcome to Ethiopia's fly factory. Science. 2007;317(5836):310-3.

18. ADF. Ethiopia - CSTT Project completion report. Report from the African Development Fund, 2014.

19. Bekele J, Asmare K, Abebe G, Ayelet G, Gelaye E. Evaluation of Deltamethrin applications in the control of tsetse and trypanosomosis in the Southern Rift Valley areas of Ethiopia. Vet Parasitol. 2010;168(3–4):177-84.

20. Alemu T, Kapitano B, Mekonnen S, Aboset G, Kiflom M, Bancha B, et al. Area-wide control of tsetse and trypanosomosis: Ethiopian experience in the Southern Rift Valley. In: Vreysen M, Robinson A, Hendrichs J, editors. Area-Wide Control of Insect Pests: Springer; 2007. p. 325-35.

21. Girmay G, Arega B, Tesfaye D, Berkvens D, Muleta G, Asefa G. Community-based tsetse fly control significantly reduces fly density and trypanosomosis prevalence in Metekel Zone, Northwest, Ethiopia. Trop Anim Health Pro. 2016;48(3):633-42.
