# Supplementary material for: Past and Ongoing Tsetse and Animal Trypanosomiasis Control Operations in Five African Countries: A Systematic Review
Source: PLoS Negl Trop Dis. 2016 Dec 27;10(12):e0005247. doi: 10.1371/journal.pntd.0005247 (PMC5222520; doi:10.1371/journal.pntd.0005247)
Supplement: S4 Table — (DOCX) [file pntd.0005247.s006.docx]

**S4 Table. Detailed description of six well-documented control operations implemented in Uganda since 1980**

Abbreviations:

- General: AAT, Animal African Trypanosomiasis; FITCA, Farming in Tsetse Controlled Areas; HAT, Human African Trypanosomiasis; LHRI, Livestock Health Research Institute; SOS, Stamping Out Sleeping Sickness; T&T, tsetse and trypanosomiasis; WHO, World Health Organisation
- Tsetse species: GFF, *Glossina fuscipes fuscipes*; GP, *G. pallipides*
- Interventions: DA, diminazene aceturate; DLT, deltamethrin; ISM, isometamidium salt; ITC, insecticide-treated cattle; ITT, insecticide-impregnated traps and/or targets; TRY, use of trypanocidal drugs

| **Project** | **Government work, 1988 onwards, Busoga** |
| --- | --- |
| **Objectives** | Control of HAT epidemic |
| **Interventions** | - ITT: DLT impregnated traps, 10/km^2^ - ITC: in some areas, DLT pour-on |
| **Location** | 15 sub-counties in the Busoga region |
| **Surface of target area** | ~ 2,200 km^2^ |
| **Initial target population** | Humans |
| **Tsetse species** | *GFF* |
| **Trypanosome species** | *T. brucei rhodesiense, T. vivax* |
| **Budget** | 300,000 USD per year (=136 USD / km^2^ / year) |
| **Funders** | French and Ugandan Governments, European Commission and WHO |
| **Year starting** | 1988 |
| **Duration of project** | At least 3 years |
| **Collaborators & implementers** | Tsetse Control Department |
| **Involvement of community** | Information campaigns, assistants recruited within community to maintain the traps. |
| **Deviations, set-backs and difficulties** | Elimination not possible because of the persistent re-invasion from infested areas. |
| **Outcome measurement** | 99% reduction in tsetse density (100% in some parishes). Reduction of human cases, to nil in some parishes. |
| **Progress against the objectives** | Success |
| **Sustainability** | Not reported. However, the sustainability of the control of HAT foci in a neighbouring district (Tororo) was reported. The outbreaks were managed using a combination of mass treatment of cattle and pigs with deltamethrin and trypanocides. A 70% decrease in AAT prevalence was reported in cattle. No further human cases were reported after the intervention and for up to 17 years. |
| **References** | [1-3] |

| **Project** | **LHRI work, 1991-1993, Busia** |
| --- | --- |
| **Objectives** | Control of AAT |
| **Interventions** | - ITC: DLT pour-on in cattle: initially 23/km^2^ subsequently 2/km^2^ |
| **Location** | Busia district in the East region |
| **Surface of target area** | 130 km^2^ (4 parishes) |
| **Initial target population** | 1,000 cattle |
| **Tsetse species** | *GFF* |
| **Trypanosome species** | *T. brucei, T. congolense, T. vivax* |
| **Budget** | Not reported |
| **Funders** | United Nations, WHO |
| **Year starting** | 1991 |
| **Duration of project** | 2 years |
| **Collaborators & implementers** | LHRI |
| **Involvement of community** | Not reported |
| **Deviations, set-backs and difficulties** | Treatment of only 10% of cattle controlled tsetse flies but was not sufficient to keep AAT at low levels (fluctuating between 8.2 and 26.8%) |
| **Outcome measurement** | 98.4% reduction in tsetse density. 92% reduction in AAT prevalence when all cattle were treated. |
| **Progress against the objectives** | Good but 10% coverage rate of ITC was not sufficient to control AAT. |
| **Sustainability** | Not reported |
| **References** | [4, 5] |

| **Project** | **LHRI work, 1991 onwards, Tororo** |
| --- | --- |
| **Objectives** | Integrated disease control for AAT and HAT |
| **Interventions** | - ITT: DLT-impregnated traps, 4-10/km^2^ - ITC - TRY: DA mass treatments |
| **Location** | Tororo district in the East region |
| **Surface of target area** | 1,350 km^2^ |
| **Initial target population** | 50,000 cattle |
| **Tsetse species** | *GFF* |
| **Trypanosome species** | *T. brucei, T. vivax, T. congolense* |
| **Budget** | Not reported |
| **Funders** | African Union and European Union |
| **Year starting** | 1991 |
| **Duration of project** | 4 years (initial funding) but extended |
| **Collaborators & implementers** | LHRI |
| **Involvement of community** | Information only |
| **Deviations, set-backs and difficulties** | Transmission of *T. vivax* was less affected by control (shorter lifecycle, mechanical transmission) |
| **Outcome measurement** | 99.5% reduction in tsetse density and 94% reduction in AAT prevalence under the most intensive regimen (initial ITC+ITT+DA). |
| **Progress against the objectives** | Success |
| **Sustainability** | When funding came to an end, the community and government had to provide funds and labour to support the operations. However, some control operations continued and the prevalence of AAT in 2000 was lower in areas with control than in areas without control. |
| **References** | [6, 7] |

| **Project** | **FITCA programme, 1999-2004, Busoga** |
| --- | --- |
| **Objectives** | Community-based tsetse control and poverty alleviation |
| **Interventions** | - ITT: pyramidal traps - ITC - Protected zero grazing dairy units |
| **Location** | 12 districts in the Busoga region |
| **Surface of target area** | 52,000 km^2^ |
| **Initial target population** | 900,000 cattle |
| **Tsetse species** | *GFF* |
| **Trypanosome species** | *T. brucei, T. congolense, T. vivax* |
| **Budget** | 5 million USD (= 96 USD/km^2^) |
| **Funders** | European Commission |
| **Year starting** | 1999 |
| **Duration of project** | 5 years |
| **Collaborators & implementers** | African Union – Inter-African Bureau for Animal Resources, FITCA team and Government services |
| **Involvement of community** | Contribution in labour and treatment fee. Creation of farmers groups committed to insecticide treatments. Training of community assistants for ITT management. |
| **Deviations, set-backs and difficulties** | Low levels of coverage. Farmers’ groups did not persist in time due to poor management, lack of training and financial issues. |
| **Outcome measurement** | 75 to 90% reduction in tsetse density in mainland areas. |
| **Progress against the objectives** | Insufficient reduction of T&T, extension of HAT endemic area continued. Insufficient reduction of AAT was reported, however it was not a primary objective of the programme. |
| **Sustainability** | A PATTEC survey in 2009 showed that the tsetse levels had returned to pre-control levels. |
| **References** | [8-12] |

| **Project** | **Current PATTEC campaign** |
| --- | --- |
| **Objectives** | Elimination of tsetse |
| **Interventions** | - ITC - TRY - SIT |
| **Location** | Area around Lake Kyoga in the South-East |
| **Surface of target area** | 21,000 km^2^ |
| **Initial target population** | 910,000 cattle |
| **Tsetse species** | *GFF, GP* |
| **Trypanosome species** | *T. brucei, T. congolense, T. vivax* |
| **Budget** | 10 million USD (Phase I) (= 476 USD/km^2^) |
| **Funders** | African Development Fund |
| **Year starting** | 2005 |
| **Duration of project** | 6 years (Phase I) |
| **Collaborators & implementers** | Uganda Trypanosomiasis Control Council |
| **Involvement of community** | Information only. Stronger involvement within suppression phase and responsibility of community for barrier maintenance was planned. |
| **Deviations, set-backs and difficulties** | Failure of Ethiopia’s factory to provide the irradiated flies for the SIT operations. Re-orientation towards aerial spraying but due to poor project management, the elimination phase was not implemented. |
| **Outcome measurement** | Tsetse suppression of 50 to 75% was achieved in a 12,000 km^2^ area. |
| **Progress against the objectives** | Elimination not achieved. |
| **Sustainability** | As the funding came to an end in 2011, the control activities stopped. No barriers were maintained so reinvasion may have occurred. |
| **References** | [13-16] |

| **Project** | **SOS campaign, 2006 onwards, North and East** |
| --- | --- |
| **Objectives** | Control of HAT epidemics through management of animal reservoir |
| **Interventions** | - TRY: mass treatment of cattle with DA or ISM - ITC: DLT restricted spraying monthly 20/km^2^ |
| **Location** | Districts of Amolotar, Apac, Dokolo and Lira in the North region + district of Kaberamaido in the East region (i.e. Northern limit of the repartition of *T.b. rhodesiense*) |
| **Surface of target area** | 8,800 km^2^ |
| **Initial target population** | 180,000 cattle treated with trypanocides |
| **Tsetse species** | *GFF* |
| **Trypanosome species** | *T. brucei rhodesiense* |
| **Budget** | Not reported |
| **Funders** | Private companies (CEVA Santé Animale, Industri Kapital) |
| **Year starting** | 2006 |
| **Duration of project** | Ongoing |
| **Collaborators & implementers** | Makerere University (with inputs from Edinburgh University and Uganda Trypanosomiasis Control Council) |
| **Involvement of community** | The community contributed to the costs of the treatment and provided labour. However the mobilisation was poor. |
| **Deviations, set-backs and difficulties** | The first treatments were covered by the project but the costs of the subsequent sprayings were left to the farmers (3V Vets initiative). DLT application coverage in cattle slowly decreased (from 80% to 40%), returning to pre-campaign levels by 2009.  Although a large proportion of farmers spray their livestock with acaricides as part of the SOS campaign area, almost half of them used amitraz-based products, which are not efficient against tsetse but more affordable. Immigration of untreated cattle in some districts triggered a re-treatment operation in 2008. |
| **Outcome measurement** | 75% reduction of the prevalence of AAT within the first few months. Large reduction of HAT cases. |
| **Progress against the objectives** | Low treatment coverage due to community hesitance, low rate of subsequent spraying, lack of treatments in pigs, and continuous importation of cattle from endemic areas. |
| **Sustainability** | Although the prevalence of both HAT and AAT was initially reduced, it increased again afterwards. Sustainability of control is uncertain owing to the poor mobilisation of the community. Authors suggested that a policy framework was necessary to support the programme. |
| **References** | [9, 17-20] |

**References**

1. Lancien J, Muguwa J, Lannes C, Bouvier JB. Tsetse and human trypanosomiasis challenge in south eastern Uganda. Int J Trop Insect Sci. 1990;11(03):411-6.

2. Lancien J. Lutte contre la maladie du sommeil dans le Sud-Est Ouganda par piégeage des glossines. Ann Soc Belg Med Trop. 1991;71:35-47.

3. Magona JW, Walubengo J. Mass-treatment and insecticide-spraying of animal reservoirs for emergency control of Rhodesiense sleeping sickness in Uganda. J Vector Borne Dis. 2011;48(2):105-8.

4. Okiria R, Okuna NM, Magona JW, Mayende JS. Sustainability of tsetse control by subsequent treatment of 10% of a previously treated Ugandan cattle population with 1% w/v deltamethrin. Trop Anim Health Pro. 2002;34(2):105-14.

5. Magona JW, Mayende JS, Okiria R, Okuna NM. Protective efficacy of isometamidium chloride and diminazene aceturate against natural Trypanosoma brucei, Trypanosoma congolense and Trypanosoma vivax infections in cattle under a suppressed tsetse population in Uganda. Onderstepoort J Vet Res. 2004;71(3):231-7.

6. Magona J, Okuna N, Katabazi B, Omollo P, Okoth J, Mayende J, et al. Control of tsetse and animal trypanosomosis using a combination of tsetse-trapping, pour-on and chemotherapy along the Uganda-Kenya border. Rev Elev Med Vet Pays Trop. 1998;51(4):311-5.

7. Magona JW, Greiner M, Mehlitz D. Impact of tsetse control on the age-specific prevalence of trypanosomosis in village cattle in southeast Uganda. Trop Anim Health Pro. 2000;32(2):87-98.

8. Agriconsortium. End of project evaluation study for FITCA regional and national components of five countries, Kenya, Uganda, Tanzania, Rwanda and Ethiopia. Report from the Farming In Tsetse Controlled Areas project, 2005.

9. Selby RJ. Limiting the northerly advance of Trypanosoma brucei rhodesiense in post conflict Uganda. PhD thesis, University of Edinburgh. 2010.

10. Hyseni C, Kato AB, Okedi LM, Masembe C, Ouma JO, Aksoy S, et al. The population structure of Glossina fuscipes fuscipes in the Lake Victoria basin in Uganda: implications for vector control. Parasit Vectors. 2012;5:222.

11. AU-IBAR. About FITCA: African Union - Interafrican Bureau for Animal Resources; 2013 [Available from: <http://www.au-ibar.org/fitca>.

12. ADF. Multinational project - CSTT - appraisal report Report from the African Development Fund, 2004.

13. COCTU. National policy for the eradication of tsetse flies and elimination of trypanosomiasis. Report from the Coordinating Office for Control of Trypanosomiasis in Uganda - Ministry of Agriculture, Animal Industry and Fisheries., 2014.

14. Shaw A, Torr S, Waiswa C, Robinson T. Comparable costings of alternatives for dealing with tsetse: estimates for Uganda. Report from the Pro-Poor Livestock Policy Initiative, Food and Agriculture Organization of the United Nations, 2007.

15. Shaw AP, Torr SJ, Waiswa C, Cecchi G, Wint GR, Mattioli RC, et al. Estimating the costs of tsetse control options: an example for Uganda. Prev Vet Med. 2013;110(3-4):290-303.

16. ADF. Uganda - CSTT Project Completion Report. Report from the African Development Fund, 2012.

17. Kabasa JD. Public-private partnership works to stamp out sleeping sickness in Uganda. Trends Parasitol. 2007;23(5):191-2.

18. Morton J. The innovation trajectory of sleeping sickness control in Uganda: research knowledge in its context. Report from the Department for International Development, UK, 2010.

19. Bardosh K, Waiswa C, Welburn SC. Conflict of interest: use of pyrethroids and amidines against tsetse and ticks in zoonotic sleeping sickness endemic areas of Uganda. Parasit Vectors. 2013;6:204.

20. Bouyer J, Seck MT, Sall B. Misleading guidance for decision making on tsetse eradication: Response to Shaw et al. (2013). Prev Vet Med. 2013;112(3–4):443-6.
