# Supplementary material for: Past and Ongoing Tsetse and Animal Trypanosomiasis Control Operations in Five African Countries: A Systematic Review
Source: PLoS Negl Trop Dis. 2016 Dec 27;10(12):e0005247. doi: 10.1371/journal.pntd.0005247 (PMC5222520; doi:10.1371/journal.pntd.0005247)
Supplement: S5 Table — (DOCX) [file pntd.0005247.s007.docx]

# **S5 Table. Detailed description of three well-documented control operations implemented in Zambia since 1980**

Abbreviations:

- General: AAT, Animal African Trypanosomiasis; RTTCP, Regional Tsetse and Trypanosomiasis Control Programme; T&T, tsetse and trypanosomiasis
- Tsetse species: GMM, *Glossina morsitans morsitans*; GMC, *Glossina morsitans centralis*
- Interventions: DLT, deltamethrin; ITT, insecticide-impregnated traps and/or targets; SAS, sequential aerial spraying; TRY, use of trypanocidal drugs

| **Project** | **Government work, 1987-89, Western province** |
| --- | --- |
| **Objectives** | Elimination of tsetse |
| **Interventions** | - ITT: DLT-impregnated, odour-baited screens 4/km^2^ - TRY in infected cattle |
| **Location** | Senanga district in the Western province |
| **Surface of target area** | 2,000 km^2^ (pilot project) |
| **Initial target population** | 12,000 cattle |
| **Tsetse species** | *GMC* |
| **Trypanosome species** | *T. brucei, T. congolense, T. vivax* |
| **Budget** | Not reported |
| **Funders** | European Union |
| **Year starting** | 1987 |
| **Duration of project** | 3 years (pilot project) |
| **Collaborators & implementers** | Zambian Department of Veterinary and Tsetse Control Services and PAN Livestock Services (UK) |
| **Involvement of community** | Information only. Further involvement failed. |
| **Deviations, set-backs and difficulties** | The project team did not manage to involve the community in the project, especially in the long-term management of the targets. |
| **Outcome measurement** | Elimination of tsetse population was reached after 7 months of control, correlated with over 93% reduction in AAT prevalence. After a year, targets were progressively removed and used to extend the control area, however a barrier of targets was left in place. |
| **Progress against the objectives** | Success |
| **Sustainability** | Intensive monitoring of tsetse population was maintained [1]. The control area was progressively extended over the years, reaching a total surface of 11,500 km^2^ in the 2000s. This area is considered tsetse-controlled and protected from reinvasion by barriers, however the absence of flies inside the areas is not regularly monitored any longer. |
| **References** | [1-4] |

| **Project** | **RTTCP work, 1989-94, Eastern province** |
| --- | --- |
| **Objectives** | Sustainable control of tsetse, following reinvasion of tsetse into this previously cleared area |
| **Interventions** | - ITT: DLT-impregnated, odour-baited screens |
| **Location** | Petauke district in the Eastern province |
| **Surface of target area** | 300 km^2^ (later extended to 900 km^2^) |
| **Initial target population** | 2,400 cattle |
| **Tsetse species** | *GMM* |
| **Trypanosome species** | *T. vivax, T. brucei, T. congolense* |
| **Budget** | Not reported |
| **Funders** | European Union |
| **Year starting** | 1989 |
| **Duration of project** | 6 years |
| **Collaborators & implementers** | RTTCP |
| **Involvement of community** | Not involved |
| **Deviations, set-backs and difficulties** | Due to the patchy distribution of tsetse flies in that area, the deployment of the targets was restricted to tsetse-favourable habitats, leading to an overall density lower than the recommended 4 targets per km^2^. |
| **Outcome measurement** | No flies caught. 82 % reduction in AAT prevalence. |
| **Progress against the objectives** | Elimination achieved inside control area. |
| **Sustainability** | Although barriers were kept in place, reinvasion was still expected from the highly infested neighbouring areas, as well as from movements of cattle in and out the control area in search of grazing. Only the core of the area was considered truly tsetse-free. |
| **References** | [5-7] |

| **Project** | **Current PATTEC campaign** |
| --- | --- |
| **Objectives** | Elimination of tsetse |
| **Interventions** | - SAS |
| **Location** | Kwando-Zambezi Tsetse Belt (across Angola, Botswana, Namibia and Zambia) |
| **Surface of target area** | 22,000 km^2^ (from which 10,000 km^2^ had been cleared in the 1990s) |
| **Initial target population** | Not reported |
| **Tsetse species** | *GMC* |
| **Trypanosome species** | *T. congolense, T. vivax* |
| **Budget** | 3 million USD (= 250 USD/km^2^) |
| **Funders** | Government |
| **Year starting** | 2008 |
| **Duration of project** | Ongoing |
| **Collaborators & implementers** | Zambian Department of Veterinary and Tsetse Control Services and service providers of aerial spraying |
| **Involvement of community** | Information about control activities, hiring of casual labour for barrier maintenance. |
| **Deviations, set-backs and difficulties** | Slow progress of the campaign has been attributed to lack of coordination between the different members involved (PATTEC coordination office, regional project and local governments). |
| **Outcome measurement** | 100 % reduction in tsetse density and AAT prevalence. |
| **Progress against the objectives** | Success |
| **Sustainability** | Barriers in place and regular surveillance to detect potential resurgence of tsetse. A more intensive tsetse detection operation is needed to confirm whether the population has been eliminated. |
| **References** | [8] + (T&T control officer, Zambia, February 2016) |

**References**

1. Bouyer J, Seck MT, Sall B. Misleading guidance for decision making on tsetse eradication: Response to Shaw et al. (2013). Prev Vet Med. 2013;112(3–4):443-6.

2. Putt SNH, Leslie J, Willemse L. The economics of trypanosomiasis control in Western Zambia. Acta Vet Scand. 1988:394-7.

3. Willemse L. A trial of odour baited targets to control the tsetse fly, Glossina morsitans centralis (Diptera: Glossinidae) in west Zambia. Bull Entomol Res. 1991;81(3):351-7.

4. Hargrove J. Tsetse eradication: sufficiency, necessity and desirability. Report from the Department for International Development, University of Edinburgh, UK., 2003.

5. Van den Bossche P. The control of Glossina morsitans morsitans (Diptera: Glossinidae) in a settled area in Petauke District (Eastern Province, Zambia) using odour-baited targets. Onderstepoort J Vet Res. 1997;64(251-257).

6. Van den Bossche P, Doran M, Connor RJ. An analysis of trypanocidal drug use in the Eastern Province of Zambia. Acta Trop. 2000;75(2):247-58.

7. Robinson TP, Harris RS, Hopkins JS, Williams BG. An example of decision support for trypanosomiasis control using a geographical information system in eastern Zambia. Int J Geogr Inf Sci. 2002;16(4):345-60.

8. Chilongo K. Zambia and the Kwando-Zambezi tsetse eradication project. PATTEC Coordinators' meeting; Ethiopia; 2012.
